# DNA origami-engineered gold nanoparticle multimers for ultrasensitive, label-free SERS detection of small molecules and biomolecules

**DOI:** 10.1039/d6ra02441f

**Published:** 2026-04-22

**Authors:** Yaosen Wang, Yanlong Cai, Huaizhou Jin, Shangzhong Jin

**Affiliations:** a College of Optical and Electronic Technology, China Jiliang University Hangzhou 310018 China jinhuaizhou@zjut.edu.cn; b Key Laboratory of Quantum Precision Measurement, College of Physics, Zhejiang University of Technology Hangzhou China S23040803033@cjlu.edu.com

## Abstract

Surface-Enhanced Raman Spectroscopy (SERS) is a powerful vibrational spectroscopy technique capable of single-molecule sensitivity, yet its performance is critically dependent on the precise engineering of plasmonic hotspots. Conventional dimer-based substrates offer limited enhancement due to their restricted hotspot volume and intensity. Here, we overcome this limitation by leveraging the programmable addressability of DNA origami to construct a plasmonic nanofork antenna (DONA) that directs the assembly of 80 nm gold nanoparticles into well-defined multimers (trimers, tetramers, and pentamers). By systematically increasing the number of thiolated staple strands and refining the post-assembly conjugation workflow, we achieve reproducible sub-2 nm interparticle gaps, enabling dense and intense electromagnetic hotspots. This multimeric platform enables label-free SERS detection of Rhodamine 6G (R6G) down to an ultralow concentration of 10^−14^ M, with characteristic Raman peaks at 613 cm^−1^ and 1512 cm^−1^ clearly resolved. Furthermore, we demonstrate the versatility of this platform by detecting proteins (streptavidin, thioredoxin) at sub-picomolar levels. Our work establishes DNA-origami-engineered multimers as a robust and generalizable strategy for ultrasensitive, label-free biochemical sensing.

## Abstract 2

Tetrameric plasmonic hotspots from DNA origami outperform dimers, enabling ultralow-concentration SERS sensing.

## Graphical abstract

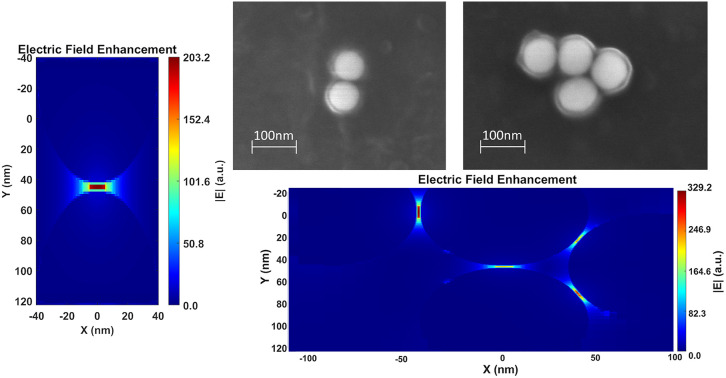

## Introduction

Surface-Enhanced Raman Spectroscopy (SERS) relies on plasmonic hotspots for sensitive detection,^1–4^ which is commonly generated by assembling gold nanoparticles (AuNPs) into dimers.^5–7^ Although dimer-based hotspots are practical for detection, they are intrinsically limited in their enhancement capability. While a 0.7–1.2 nm gap represents the sweet spot for maximum SERS enhancement (∼10^9^),^8^ higher-order multimers offer a promising alternative, generating more intense and numerous hotspots than dimers,^9–11^ thus directly surpassing the sensitivity constraints of simple dimers.

DNA origami technology presents a promising approach to address these challenges, with applications including regulating immune cell signaling^12^ and performing single-molecule detection^13–16^ (typically with labeled molecules attached to the structure directly) to facilitate fluorescence studies,^17,18^ investigating the molecular states and spin crossover of hemoglobin,^19^ and constructing dynamic DNA tweezers^20^ and sensors.^21^ Its unparalleled spatial addressability^22^ allows for the precise organization of components into diverse nanostructures with programmable motion.^23,24^ In the field of nanophotonics, this capability has been exploited to create precise assemblies such as AuNP dimers (including bowtie^25^ and nanostar^26^ configurations) for plasmonic enhancement. Pioneering work by Kosti Tapio^27–29^*et al.* demonstrated this potential through a DNA origami nanofork antenna (DONA), which enabled the reproducible creation of fixed, narrow nanogaps, overcoming the limitations of stochastic chemical methods.

Unlike previous DNA-origami SERS platforms that pre-functionalize Raman reporters at defined hotspot locations—restricting detection to known targets—our system relies on the free adsorption of target molecules onto AuNP surfaces, making it more adaptable for diverse analytes. This label-free mode also complicates theoretical modeling, as conventional FDTD simulations assume fixed molecular positions—an assumption that does not reflect the random adsorption and diffusion in our experiments.

Accordingly, to advance the performance of label-free SERS detection, we selected Rhodamine 6G (R6G), a small dye molecule, as our test subject to benchmark the detection capability. We systematically refined the classic DNA origami nanofork antenna (DONA) architecture. First, 80 nm AuNPs were employed instead of 60 nm ones to ensure LSPR resonance matching with the 638 nm laser,^30^ generating stronger electromagnetic field enhancement in the assembled multimers. Second, the preparation workflow was refined to ensure a high yield of target structures by first assembling the DNA origami scaffold independently before attaching the AuNPs, a strategy that enhances structural fidelity. Crucially, by strategically increasing the number of attachment sites, we promoted the formation of higher-order multimers (trimers, tetramers) rather than simple dimers. These multimers provide a significantly increased number and density of plasmonic hotspots compared to dimers. More importantly, the complex coupling in a multimer can generate synergistic electromagnetic field enhancement, creating substantially higher SERS intensities at the junction sites. For micro-molecules, this configuration also increases the probability of residing within a high-field region, thereby improving the detection sensitivity and reliability. Ultimately, our optimized DONA substrate demonstrated its capability through the label-free identification of R6G^31,32^ at an ultralow concentration of 10^−14^ M, with its characteristic Raman fingerprints clearly discernible. This verifies the advantage of combining larger nanoparticles as multimers for superior SERS enhancement, establishing a robust strategy for highly sensitive sensing platforms concerning label-free detection.

## Experimental

### Materials for AuNP

To synthesize AuNP, CTAC, HAuCl_4_·3H_2_O, NaBH_4_, ascorbic acid, and sodium hypochlorite solution are required as materials. All reagents mentioned above were obtained from domestic commercial suppliers in China, including Adamas-beta, Macklin, and Aladdin.

### Seeded growth of 80 nm AuNP

**Fig. 1:**
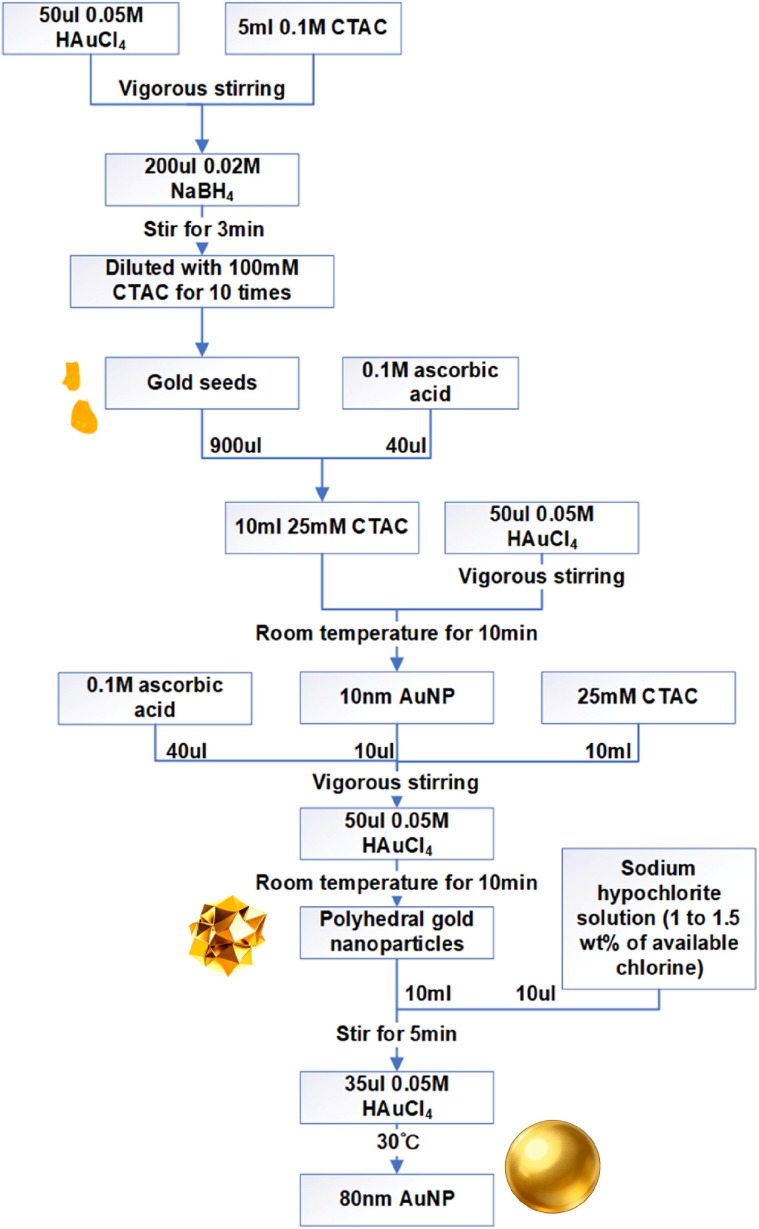
Process of producing 80 nm AuNP.

The whole procedure is shown in Fig. 1. At room temperature, 50 µL of 0.05 M chloroauric acid solution was added to 5 mL of 0.1 M CTAC solution, followed by rapid injection of 200 µL of freshly prepared 0.02 M sodium borohydride solution under vigorous stirring. After 3 minutes of reaction, the mixture was diluted with CTAC to obtain 1–3 nm gold seeds. Subsequently, 10 µL of these gold seeds and ascorbic acid were introduced into 10 mL of growth solution, followed by the addition of 50 µL of 0.05 M chloroauric acid. The system was left undisturbed for growth, yielding roughly faceted gold particles. Finally, oxidative etching was performed by sequentially adding 10 µL of NaClO solution and 35 µL of 0.05 M chloroauric acid, followed by incubation at 30 °C to ultimately produce around 80 nm gold nanospheres.^33^

### Materials for DNA origami

The DNA origami structure was constructed using 202 staple strands (including four thiol-modified strands) and the M13mp18 scaffold strand. The staple strands were purchased from Sangon Biotech (Shanghai) Co., Ltd, and the M13mp18 scaffold was obtained from New England Biolabs. Gold nanoparticles (AuNPs) with a diameter of 80 nm were prepared. The buffers, including 1× TAE buffer and 10× TAE buffer with varying Mg^2+^ concentrations, were prepared using TAE powder from Beyotime Biotechnology (Shanghai), MgCl_2_ from Macklin Inc (Shanghai), and NaBr from Aladdin (Shanghai). Sodium dodecyl sulfate (SDS) was sourced from Sigma-Aldrich. All solutions were prepared with ultrapure water.

### Assembly of DNA origami

**Fig. 2:**
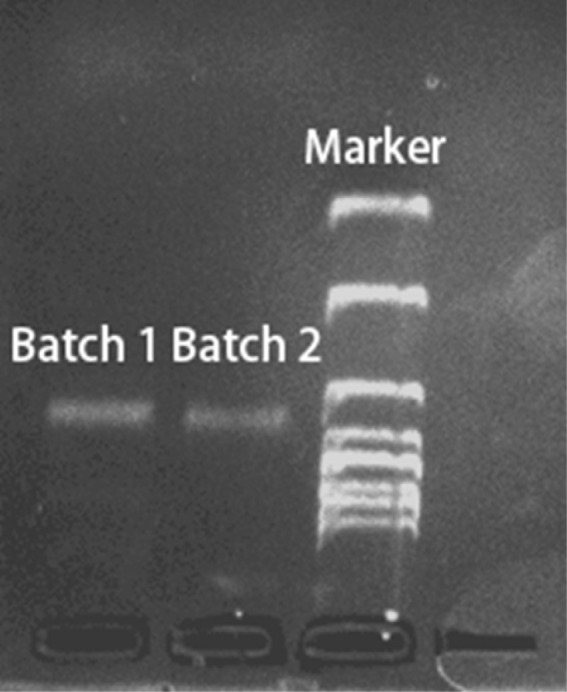
Gel electrophoresis of two independent DONA batches. Under UV illumination, they reveal consistent migration patterns, demonstrating high structural uniformity and excellent experimental reproducibility.

The staple strands were mixed and vortexed to form a staple strand solution, which was then diluted to 1% concentration, corresponding to 1 µM per staple strand. A 20 µL aliquot of the diluted staple solution was combined with 67.5 µL of ultrapure water, 10 µL of 10× TAE buffer containing 0.1 M Mg^2+^, and 2.5 µL of M13mp18 scaffold strand in a 0.2 mL PCR tube (the concentration of each staple strand was 20 times that of the M13mp18 scaffold strand, thereby ensuring the integrity of the assembly). The mixture was vortexed thoroughly and subjected to an annealing program starting at 80 °C and decreasing to 20 °C, with a holding time of 30 minutes at each 2 °C step. After annealing, the DNA origami product was purified *via* agarose gel electrophoresis. A 1% agarose gel was prepared by dissolving 0.5 g of agarose in 50 mL of 1× TAE buffer containing 0.1 M Mg^2+^. Electrophoresis was carried out in 1× TAE buffer at a constant voltage of 80 V for 60 minutes. The gel image (Fig. 2) was acquired using a Tanon 4600SF UV gel imaging system (Tanon Science & Technology Co., Ltd).

Notably, if the gel electrophoresis purification is skipped and the crude DNA origami mixture is used directly for salt-aging, the free thiol-modified strands in the solution can increase the probability of multimer formation. Any unbound excess staple strands are removed during the following centrifugation steps, reducing background interference from the DONA structure itself and thus benefiting R6G detection.

### Conjugation of gold nanoparticles to DNA origami

The conjugation of AuNPs to DNA origami was performed after the DNA origami was assembled. After electrophoresis, the DNA origami product was extracted and purified using a SanPrep Column DNA Gel Extraction Kit, yielding 25 µL of DNA origami solution. The purified product was treated with 5 µL of 0.1 M TCEP and incubated at room temperature for 10 minutes. Meanwhile, 100 µL of AuNP solution was centrifuged at 2900 g for 5 minutes. The supernatant was removed, and the pellet was treated with 3.5 µL of 0.2% SDS, then mixed with the DNA origami solution. Finally, the mixture was incubated at 40 °C for 1 hour. RR.

**Fig. 3:**
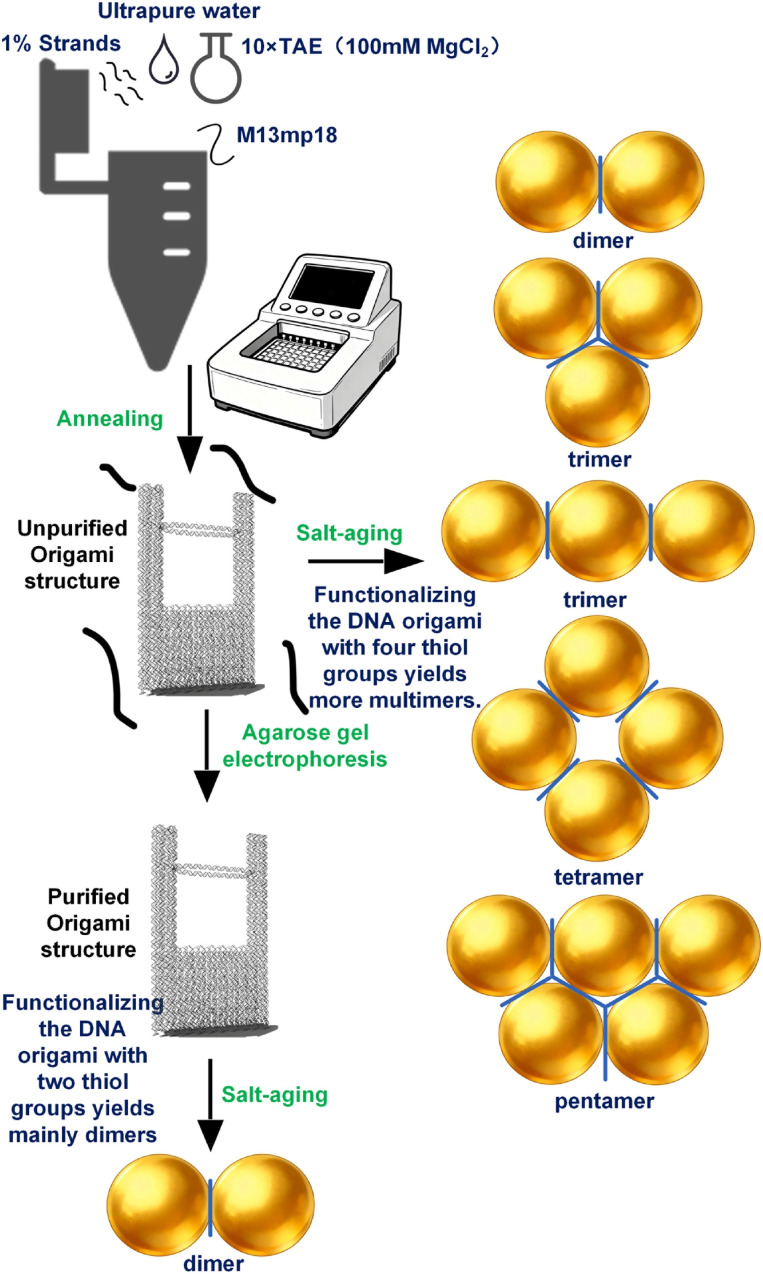
Fabrication workflow of multimers. For structures with four thiolated strands, the strategy of using the crude DNA origami mixture without prior purification during salt-aging markedly enhanced the multimer yield. Two thiolated strands origami after electrophoresis produces mainly dimers.

Sodium chloride (NaCl) facilitates the hybridization and crosslinking of surface-bound thiolated DNA by charge screening, leading to reversible nanoparticle aggregation.^34^ Meanwhile, Br^−^ ions exhibit a stronger affinity for the gold surface compared to Cl^−^, allowing for more precise control over the surface chemistry.^35^ We therefore employed salt-aging method with NaBr to promote AuNP–DNA conjugation. The principle of thiol groups linking to gold nanoparticles lies in the formation of a strong and specific Au–S chemical bond between sulfur atoms and gold atoms. Different volumes of NaBr solution were added at 10 minutes intervals as follows: three additions of 2 µL of 0.4 M NaBr, one addition of 2 µL of 1 M NaBr, two additions of 2.5 µL of 1 M NaBr, and two additions of 5 µL of 1 M NaBr. After the final NaBr addition, the mixture was incubated for another 10 minutes, followed by two additions of 7 µL of 1× TAE buffer (containing 5 mM MgCl_2_) at 10 minutes intervals. The solution was then centrifuged at 2900 g for 5 minutes. The supernatant was discarded, and the pellet was resuspended in 20 µL of 0.02% SDS and 20 µL of 1× TAE buffer (with 5 mM MgCl_2_). After another centrifugation step, the pellet was washed twice with 20 µL of 1× TAE buffer (with 5 mM MgCl_2_) and stored at 4 °C. The whole procedure is shown in Fig. 3.

To optimize cost-efficiency and structural precision, our protocol adopts a sequential two-phase assembly: first constructing the complete DNA origami structure, followed by site-specific conjugation of AuNPs. This approach is designed to overcome a key limitation of conventional methods, in which pre-conjugating AuNPs with excess thiolated DNA strands results in a dense, non-specific shell of unproductive oligonucleotides around the nanoparticle surface. This crowded interface can sterically hinder the accessibility of origami binding sites and suppress the programmed formation of higher-order multimeric structures. By reversing the order, our method significantly reduces the consumption of costly thiol-modified DNA strands while ensuring efficient hybridization at designated origami attachment points, thereby improving both yield and structural fidelity.

### SERS detection

**Fig. 4:**
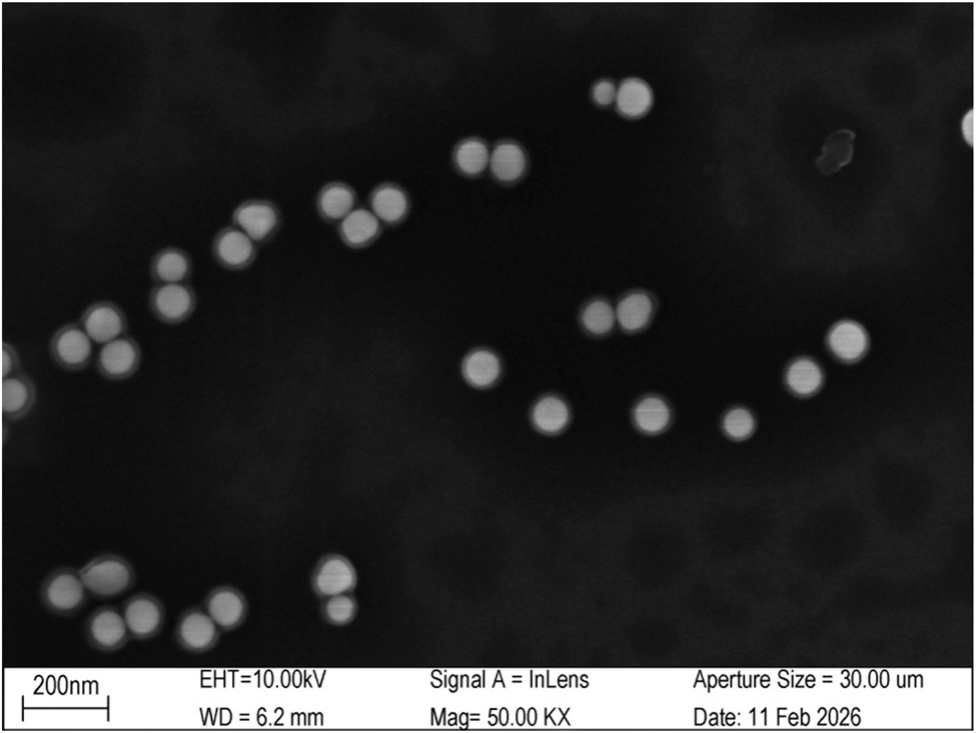
SEM image of discrete DONA multimers after oxygen plasma treatment and spin-coating with 50 mM MgCl_2_. The images were acquired by Shiyanjia Lab (https://www.shiyanjia.com/).

For SERS measurements, a silicon wafer was used as the substrate. A 2.5 µL aliquot of the DONA solution was drop-cast and air-dried for 2 hours, followed by the application and drying of 5 µL of R6G solution under the same conditions. Raman mapping was performed using a XploRA Nano Raman spectrometer (HORIBA FRANCE SAS) and preferentially directed at regions with prominent black aggregates (Fig. 4(a)), which indicate dense DONA assemblies and thus strong SERS signals. We employed a 638 nm laser 50× long working distance objective, NA = 0.50, WD = 10.6 mm for excitation. Given the high signal intensity in these areas, the laser power was carefully attenuated to 3.5% of its maximum output (1.18 mW), and integration times were kept short (1–1.5 seconds) to prevent CCD saturation and minimize photodegradation,^36^ this approach successfully secured high-fidelity spectra without compromising the substrate.

To mitigate the aggregation of DONA structures, we implemented a dispersion protocol involving oxygen plasma treatment followed by spin-coating with 50 mM MgCl_2_. The plasma treatment was performed using a CY-PIC-40K-2L plasma cleaner (Zhengzhou Chengyue Scientific Instrument Co., Ltd), and spin-coating was carried out on an EZ4-S-PP compact spin coater (Jiangsu Leibo Scientific Instrument Co., Ltd) with the following program: 400 rpm for 60 s (acceleration: 100 rpm), 1000 rpm for 100 s (acceleration: 100 rpm), and finally 1600 rpm for 60 s (acceleration: 200 rpm). SEM imaging reveals discrete DONA structures (Fig. 4).

## Results and discussion

### Design strategies for thiolated strand placement

**Fig. 5:**
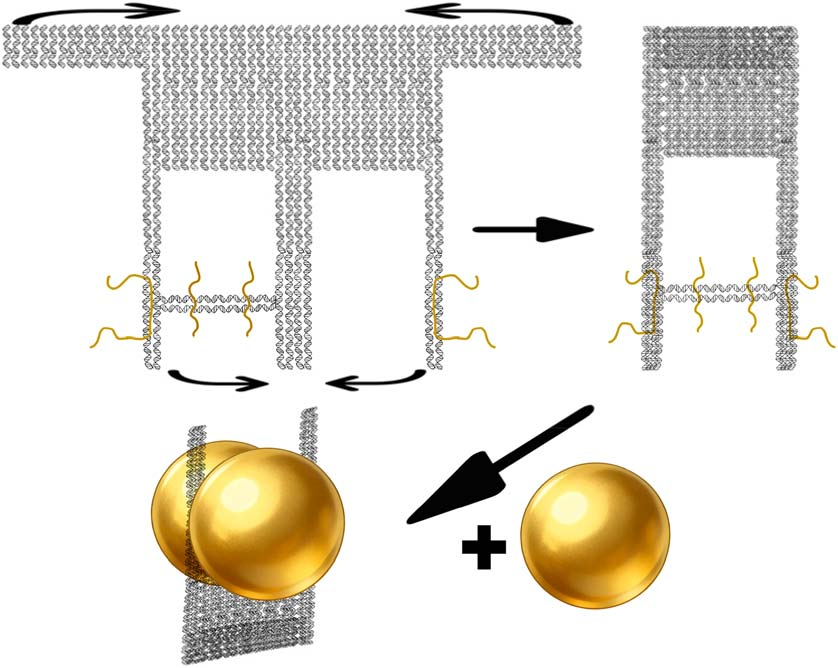
Schematic of the DONA framework. The top and bottom flaps are folded inward toward the center. The yellow strands represent thiolated strands, which would connect gold nanoparticles afterward.

The DNA origami structure is illustrated in the schematic diagram (Fig. 5). We designed two distinct strategies for positioning the thiolated strands. The first involved placing two dual-thiol-modified strands at the bridge region. The second strategy, building upon the first, added two more dual-thiol strands on both lateral sides of the structure.

### Batch-to-batch variation and its origin

**Fig. 6:**
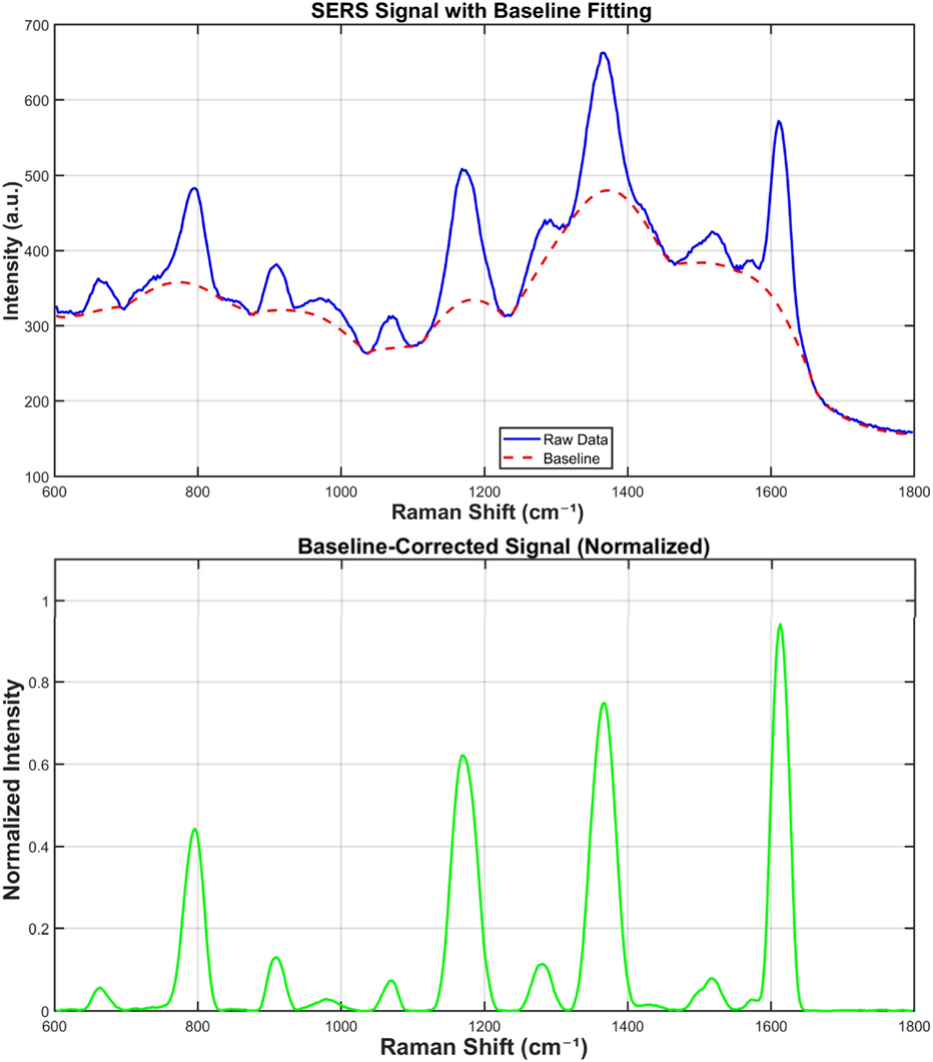
Comparison of spectra before and after baseline correction.

Owing to the programmable nature of DNA origami, such structures can be produced in batches. However, minor structural variations around the hotspots may lead to significant differences in SERS signals. To evaluate the reproducibility of the prepared samples, the SERS data obtained from two independent batches were processed as follows. The fluorescence background in the Raman spectra was automatically and accurately estimated and subtracted using an iterative morphological baseline correction method^37^ (Fig. 6).

This approach employed a morphological AVG operation—averaging the results of morphological “opening” (to suppress peaks) and “closing” (to fill valleys)—combined with softening to smooth the baseline (structuring element length = 91). The baseline-corrected spectra were then smoothed using a Savitzky–Golay filter (third-order polynomial) with a smoothing window span of 10. Peaks were identified based on appropriately set thresholds for minimum prominence, distance, height, and signal-to-noise ratio.

**Fig. 7:**
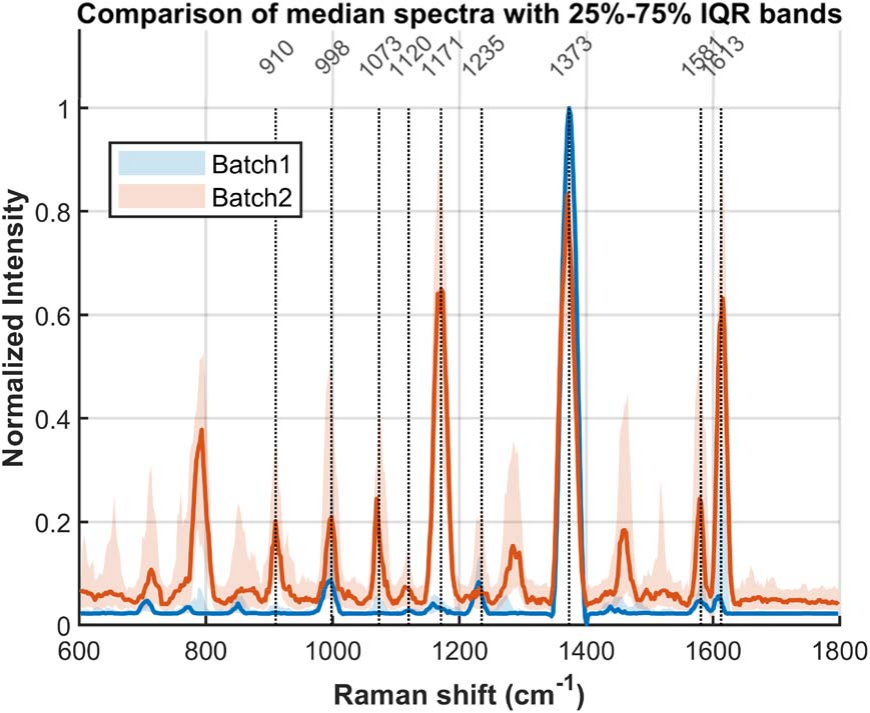
Comparison of average SERS signals between two batches with 25–75% Interquartile Range (IQR). The difference may originate from inhomogeneities in the salt-aging process.

According to Fig. 7, the SERS mapping results from both batches demonstrated high consistency in spatial distribution and highly similar spectral profiles. Batch 1 exhibited smaller signal fluctuations with a mean variance of 0.003076, whereas batch 2 showed greater variability with a mean variance of 0.021337. Although the SERS signals from the two batches were not identical, this can be attributed to the salt-aging process. During this step, subtle inconsistencies in pipetting, timing of reagent addition, portion of resuspension, or mixing efficiency can lead to variations in the resulting nanogap sizes, AuNP orientations, and the multimer-to-dimer ratio, which ultimately account for the observed differences in SERS signals. Signal differences across batches are commonly observed in DNA origami assembled SERS substrates.

Based on our experimental results, the observed SERS signals originate from multiple distinct sources. The primary contributions from the DNA origami structure are identified as follows: the peak at 1073 cm^−1^ is assigned to the symmetric stretching vibration of the PO_2_^−^ group in the phosphate-diester backbone; the signal at 1120 cm-1 is attributed to the C–O stretching vibration of the deoxyribose sugar; and the band at 1235 cm^−1^ arises from ring breathing and C–N stretching modes of the nucleobases, particularly adenine and guanine. The peaks at 1581 and 1613 cm^−1^ are assigned to the ring breathing modes coupled with C=C and C=N stretching vibrations of the purine bases, guanine (G) and adenine (A). A set of characteristic peaks at 910, 998, 1171, 1373 cm^−1^ is consistently observed; these are attributed to residues from the gold nanoparticle synthesis and to components of the buffer system.

Given inevitable batch-to-batch signal differences, we recommend performing a batch-specific calibration prior to analyte introduction. Specifically, when DNA strands are positioned within plasmonic hotspots, the intrinsic SERS background of the origami structure itself should be thoroughly characterized before introducing any external analyte.

For the control sample prepared by mixing gold nanoparticles with unassembled DNA origami components (without specific linkage), the observed SERS signals contain only peaks characteristic of the silicon substrate. This indicates the absence of non-specific plasmonic hotspots, thereby supporting the successful and intact assembly of the target DNA origami structures in our primary experiments.

### Characterization of multimers

**Fig. 8:**
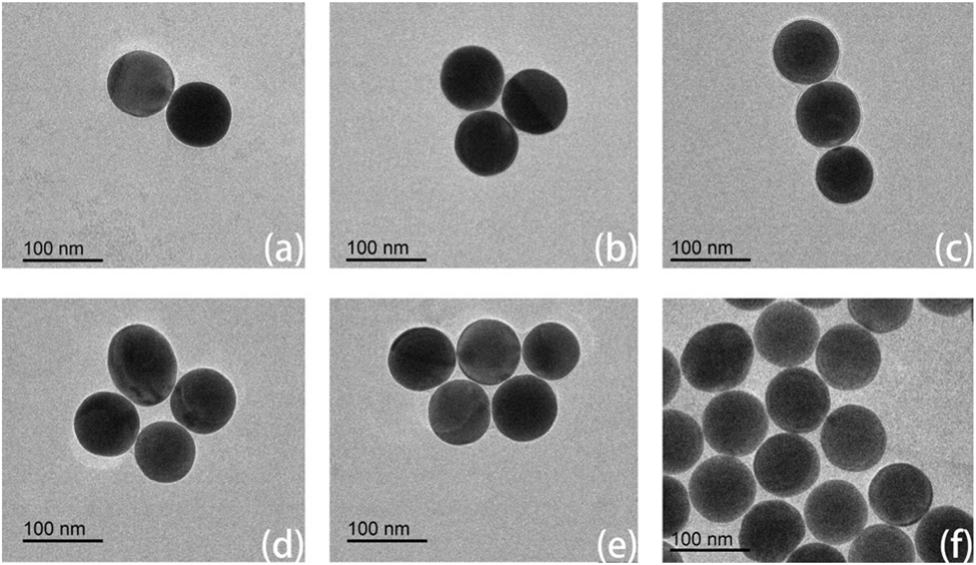
Representative TEM images of DONA structures exhibiting dimeric (a), trimeric (b and c), tetrameric (d), pentameric (e) and complex assemblies (f). Typical cases have been selected here for illustration. Due to the inherent characteristics of biological samples, DNA exhibits extremely low contrast under the high accelerating voltage of transmission electron microscopy and scanning electron microscopy, rendering it invisible. Even when negative staining with phosphotungstic acid (PTA) is applied, the DNA remains indistinguishable under high voltage.

For TEM characterization, samples were deposited onto 200 mesh copper grids, air-dried, and negatively stained with phosphotungstic acid. Imaging was performed using an SU8010 TEM instrument (FEI Company, USA). Under high-voltage imaging conditions, while the DNA origami structure itself was not directly resolvable, the nanometer gaps between AuNPs were clearly observed. Increasing the number of thiol-modified strands was found to enhance the probability of multimer formation. When only two thiol-modified strands were used, the products consisted predominantly of dimers. In contrast, the use of four thiol-modified strands led to the formation of trimers, tetramers, and even pentamers (Fig. 8). Compared to dimers, these higher-order multimers provide not only a greater number of hotspot regions but also significantly enhanced hotspot intensity, both of which are advantageous for detecting analytes at low concentrations.

**Fig. 9:**
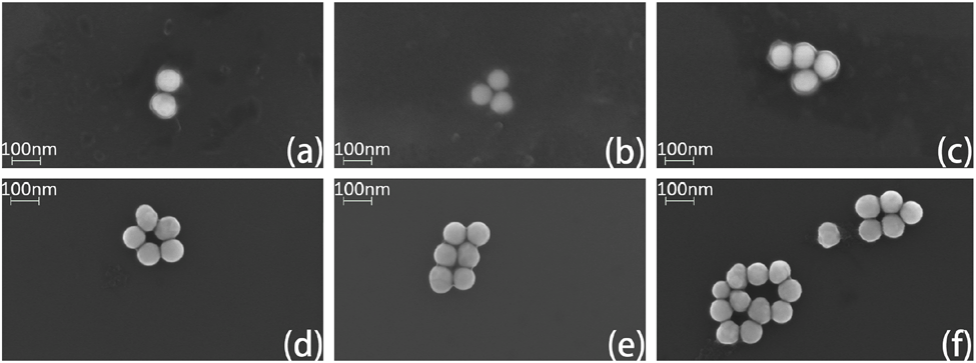
Dimer (a), trimer (b), tetramer (c), pentamer (d), hexamer (e), complex multimer (f). The morphology of the DNA origami assemblies observed by SEM is consistent with that revealed by TEM imaging.

For SEM characterization, the silicon wafer substrate was first treated with oxygen plasma, followed by spin-coating with a 50 mM MgCl_2_ solution. The sample was then applied onto the prepared substrate. Imaging was performed using a ZEISS GeminiSEM 360 (Germany). The resulting micrograph (Fig. 9), together with corresponding TEM data, confirms that the DNA origami structures successfully tether the gold nanoparticles and promote the formation of multimeric assemblies. The figure suggests interparticle gaps consistently measuring less than 2 nm. The width of gaps smaller than 2 nm is primarily governed by the thickness of the DNA origami structure.

While large-area SEM imaging is challenging due to particle clustering during sample drying—a common issue in DNA origami-templated metallization—we have addressed this by carefully selecting well-dispersed regions and performing high-resolution SEM counting on individual, distinguishable DNA origami structures. In total, 113 structures were analyzed, yielding the following distribution: 49 dimers (43.4%), 32 trimers (28.3%), 12 tetramers (10.6%), 8 pentamers (7.1%), 3 hexamers (2.7%), and 9 unresolved structures (8.0%).

### FDTD simulation of multimer enhancement

To verify the SERS performance advantage of multimers over dimers, we draw on the findings of Campione *et al.*, who reported that the resonance wavelength of linear oligomers red-shifts with increasing particle number^38^ (dimer ∼606 nm, trimer ∼625 nm, quadrumer ∼645 nm), bringing the quadrumer into better spectral overlap with the 638 nm excitation laser. Concurrently, field enhancement values increase nonlinearly (trimer 105 *vs.* dimer 92), generating stronger electromagnetic hotspots. These synergistic effects enable multimer-based SERS platforms to surpass the sensitivity limitations of simple dimers. Meanwhile, according to the FDTD simulation by Tira *et al.*,^39^ the SERS enhancement factor for dimers is approximately 2.5 × 10^8^, which increases to about 1.58 × 10^9^ for trimers, reaches a peak of around 3.98 × 10^9^ for tetramers, and then slightly decreases to approximately 2.51 × 10^9^ for pentamers.

**Fig. 10:**
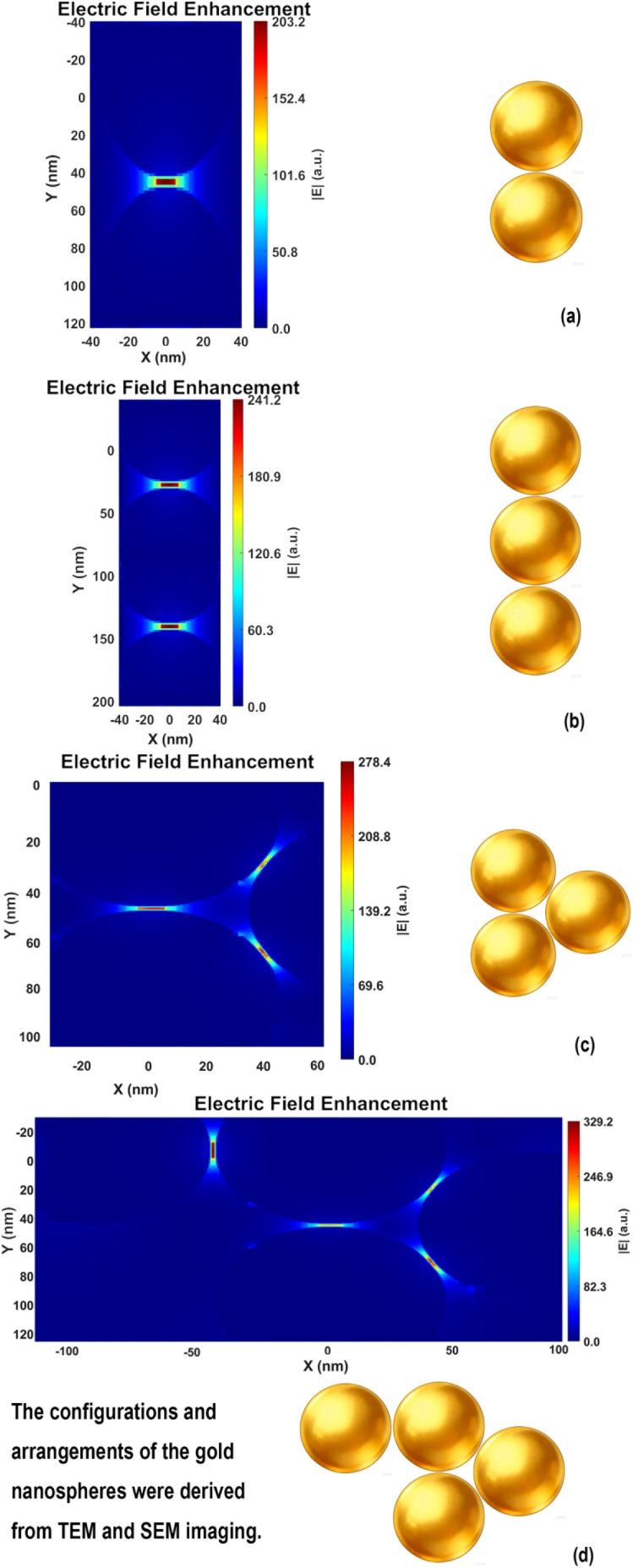
FDTD hotspot enhancement maps. (a) Dimer with a maximum enhancement of 203; (b) trimer with a maximum enhancement of 241.2; (c) trimer with a maximum enhancement of 278.4; (d) trimer with a maximum enhancement of 329.2.

We performed finite-difference time-domain (FDTD) simulations under excitation at 638 nm with a polarization angle of 90°, setting the gap between adjacent gold nanospheres to 1.5 nm. For the dimer configuration shown in Fig. 8(a) and 9(a), the maximum hotspot enhancement is approximately 203 Fig. 10(a). The trimer configuration in Fig. 8(b) and 9(b) exhibits a higher enhancement of about 278 Fig. 10(b), while the trimer arrangement in Fig. 8(c) yields a maximum enhancement of around 241 Fig. 10(c). The tetramer configuration in Fig. 9(c) shows the highest enhancement among the simulated structures, reaching approximately 329 Fig. 10(d). Using the relation that the enhancement factor (EF) scales with the fourth power of the field enhancement,^40,41^ the corresponding EF values are calculated to be 1.7 × 10^9^, 6.0 × 10^9^, 3.4 × 10^9^, and 1.2 × 10^10^, respectively. These results indicate that multimers exhibit significantly stronger enhancement compared to dimers, a finding consistent with previous reports. It should be noted, however, that the simulated configurations vary in arrangement, and the excitation source cannot always be aligned optimally (*e.g.*, in terms of polarization angle or phase) to excite the hotspots. Consequently, the simulation results are intended for reference only and do not fully represent the actual detection performance of the hotspots. Moreover, in some multimer configurations, the hotspots are uniformly enhanced across multiple sites, whereas in others, a single dominant hotspot with the highest enhancement emerges.

While label-free detection often makes it difficult to precisely position target molecules at the strongest hotspot regions, FDTD simulations confirm that the proposed structure still delivers sufficient electric field enhancement for effective detection. These results further indicate that the multimers designed in this study exhibit significantly stronger signal enhancement than dimers. Additionally, the formation of such multimers is particularly favorable for label-free detection of small molecules, not only due to their enhanced SERS performance but also because they are easier to identify under the microscope during practical measurements.

### Detection of R6G

**Fig. 11:**
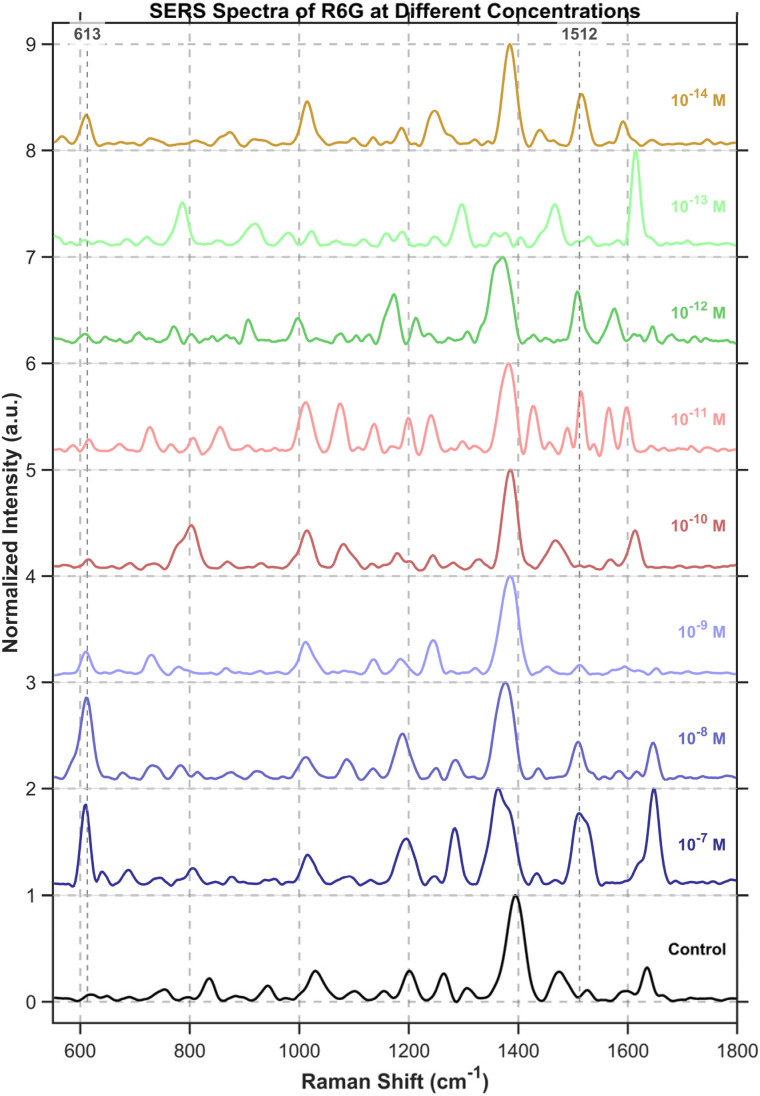
SERS signals of R6G across a concentration gradient compared with the control group. For R6G detection, the characteristic peaks at 613 cm^−1^ and 1512 cm^−1^ were chosen as indicators, as they were absent in the control (without R6G) but appeared when R6G was present. Given the random dispersion of R6G molecules across stochastic hotspots, label-free detection at ultralow concentrations requires a larger mapping area and more sampling points to locate sufficiently intense signals.

The baseline correction for the SERS spectra of R6G at each concentration was performed with an iterative morphological baseline correction algorithm, using a fixed structural element length of 31 and iterating until convergence (*λ* = 1 × 10^−4^) or a maximum of 20 iterations. To illustrate the concentration-dependent response, the spectra were vertically stacked. The figure presented was generated from samples showing the most prominent R6G signals at each concentration (Fig. 11). Since DNA origami exhibits no distinct signals at 613 cm^−1^ and 1512 cm^−1^, while R6G shows significant characteristic peaks at these two positions, we preferentially selected these two characteristic peaks to discern the presence of R6G.

Detailed detection of R6G was achieved using a custom MATLAB analysis pipeline, which specifically compared the signal intensity at characteristic R6G Raman shifts between the sample and the origami background. All spectra were first processed with an iterative morphological baseline correction algorithm, using a fixed structural element length of 51 and iterating until convergence (*λ* = 1 × 10^−4^) or a maximum of 20 iterations. The baseline-corrected spectra were subsequently smoothed with a Savitzky–Golay filter (window size: (9), polynomial order: (2)) and normalized *via* the min–max method.

For R6G identification, a set of key Raman shifts was used. The algorithm required a match of at least 3 key peaks or 1 strong peak (*e.g.*, 613, 1512, 1650 cm^−1^) within a tolerance of ±6 cm^−1^. To validate positive signals, a composite verification score was calculated from two components: peak ratio consistency (50% weight, comparing relative intensities to a benchmark) and a fingerprint match (50% weight, assessing key spectral regions and signal enhancement over the origami baseline). The suspicion score algorithm quantifies spectral similarity to R6G by calculating a weighted composite metric. This metric integrates three key components: peak matching degree (60% weight), signal enhancement level (30% weight), and spectral verification score (10% weight).

**Fig. 12:**
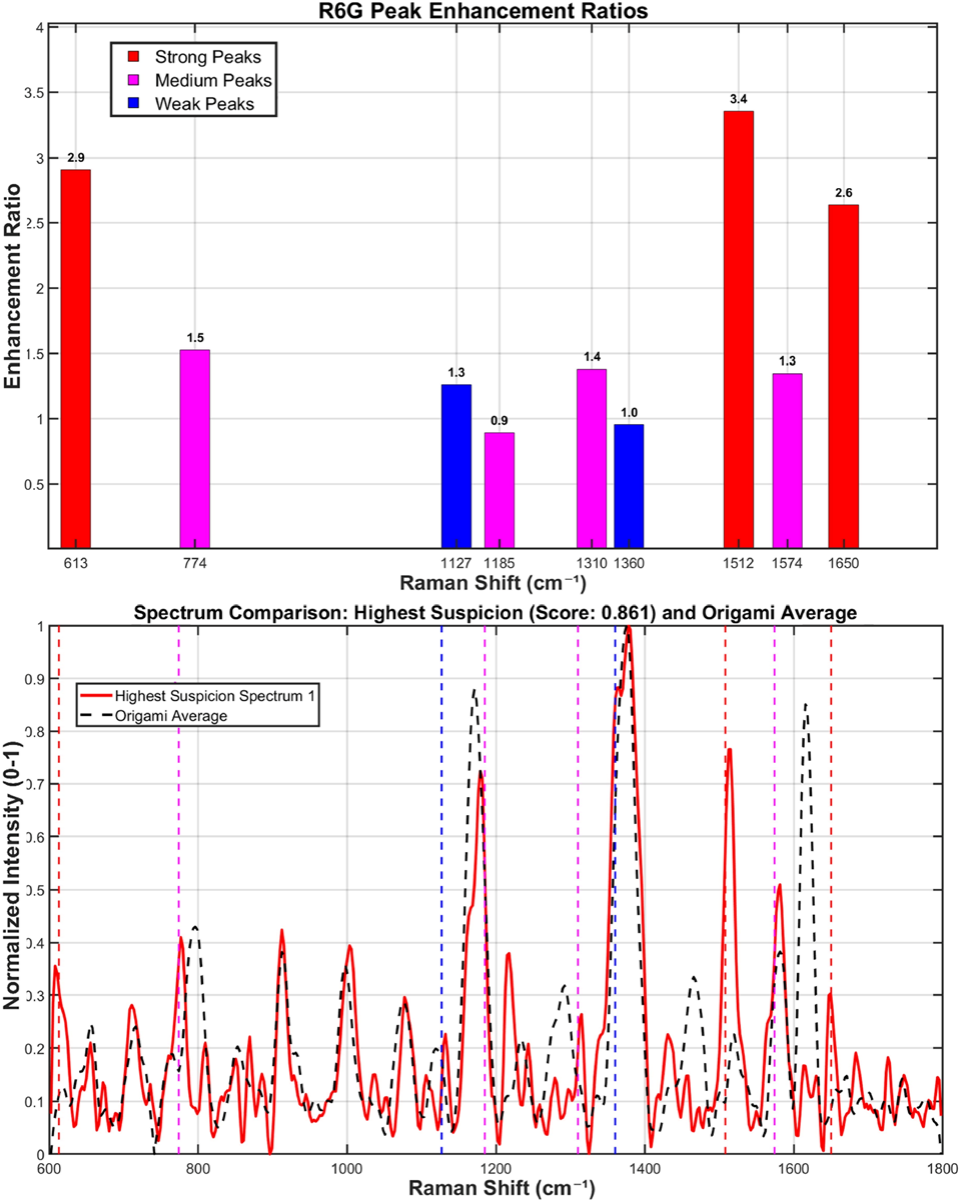
The most R6G-characteristic spectrum at a concentration of 10^−13^ M. When the analyte concentration approaches the single-molecule regime (≤10^−14^ M), the observed Raman signals originate from transiently adsorbed molecules and are inherently subject to significant photon shot noise and spectral fluctuations. Although peaks such as those at 613 cm^−1^ and 1512 cm^−1^ can be broadly identified, their poor signal-to-noise ratio renders detailed spectral interpretation unreliable. Therefore, rigorous chemometric analysis is currently limited to concentrations ≥10^−13^ M. Further methodological improvements—such as time-gated acquisition, cryogenic SERS, or statistical denoising algorithms—are required before quantitative analysis can be reliably extended into the 10^−14^ M range.

The spectrum identified as exhibiting the most characteristic R6G signatures at a concentration of 10^−13^ M showed distinct signal enhancements at key vibrational modes: a 2.9-fold increase at 613 cm^−1^, 3.4-fold at 1508 cm^−1^, and 2.6-fold at 1650 cm^−1^, relative to the origami baseline (Fig. 12).

### Detection of biotin and streptavidin

To detect biotin specifically, we positioned biotin-modified strands at the bridge region of the DNA origami. Since the biotin signal was nearly undetectable under 638 nm excitation—likely due to fluorescence interference from biomolecules—we switched to 785 nm laser 50× long working distance objective, NA = 0.50, WD = 10.6 mm excitation to suppress background fluorescence and improve signal clarity. Streptavidin from Aladdin (0.01 g ml^−1^) was diluted 200-fold into a 1× TAE buffer containing 5 mM Mg^2+^ and then mixed with the origami solution at a 9 : 1 volume ratio. The mixture was incubated at room temperature for 30 min prior to SERS measurement, which was performed using a 785 nm laser at 1.143 mW with a 2 s integration time.

**Fig. 13:**
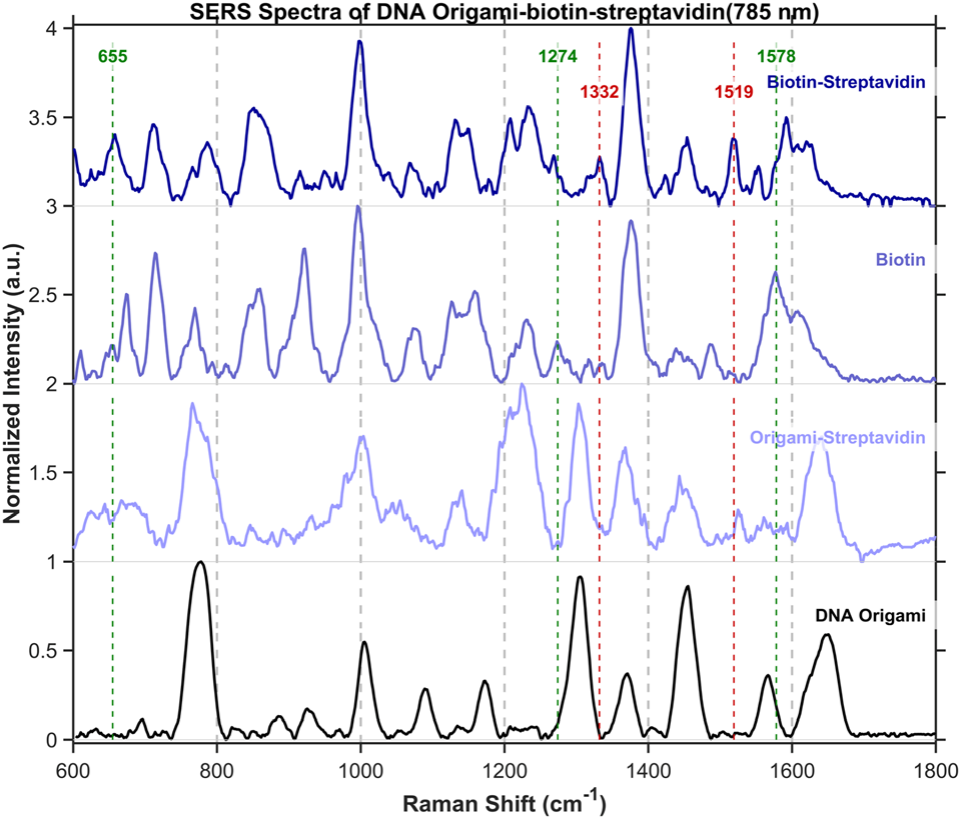
SERS spectra of DNA origami, origami–streptavidin, biotin, and biotin–streptavidin obtained under 785 nm excitation. To avoid interference from the origami substrate, characteristic peaks were assigned as follows: peaks for biotin (ureido–thiophene ring) are located at 655 cm^−1^ (C–S stretch), 1274 cm^−1^(C–N + C–C stretch of the ureido ring), and 1578 cm^−1^ (C=C/C=N stretch of the fused ring system); peaks for streptavidin (reflecting its protein structure) are observed at 1332 cm^−1^(tryptophan CH deformation) and 1519 cm^−1^ (indole ring vibration of tryptophan). In the spectra, biotin-related peaks are marked with green drop lines and streptavidin-related peaks with red drop lines. It's worth noticing that the peak at 655 cm^−1^ appears exclusively in the biotin and biotin–streptavidin groups, and is absent in both the DNA origami and origami–streptavidin controls, thereby confirming the successful incorporation of biotin into the target structures.

Control experiments were conducted using bare DNA origami structures as well as origami constructs without biotin modification that were treated with streptavidin. The experimental groups comprised biotin-modified origami and biotin-modified origami subsequently incubated with streptavidin. In the spectra presented in Fig. 13, characteristic peaks of biotin and streptavidin are marked with green and red drop lines, respectively. With sub-2-nm gaps, non-covalent binding can draw ∼5 nm streptavidin into the hotspot; this allows its detection.

### Detection of thioredoxin

**Fig. 14:**
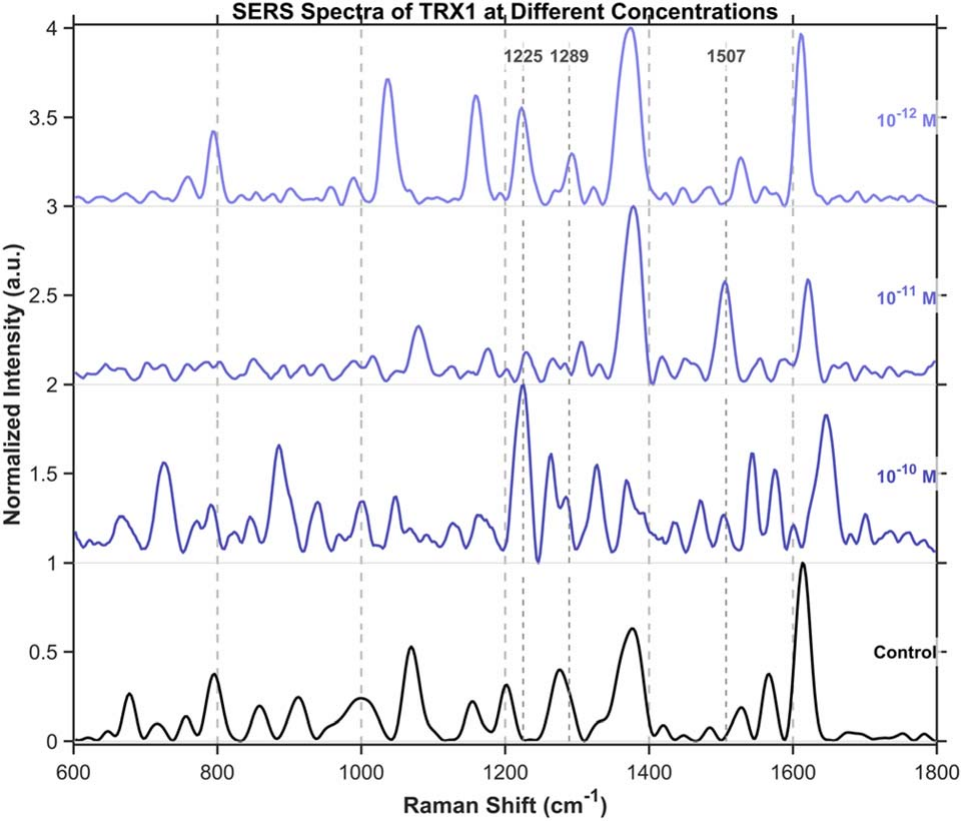
SERS spectra of thioredoxin from 10^−10^M to 10^−12^M. Thioredoxin's characteristic peaks at 1225 cm^−1^(O–H stretching, vO–H), 1289 cm^−1^ (amide III), and 1507 cm^−1^ (amide II) were used for identification. Other observed peaks were excluded from assignment due to overlap with signals from the DNA origami substrate.

To further validate the protein detection capability of DNA origami, we performed label-free analysis on thioredoxin from Hangzhou Bingrui Technology Co., Ltd (Lianke Biotech)., a protein approximately 4 nm in size. Experiment was carried out using a 638 nm laser at 3.5% power (1.18 mW) with a 1 s integration time. Even when diluted to 10^−12^ M in ultrapure water, its characteristic SERS peaks remained detectable (Fig. 14).

### Detection of amino acids

**Fig. 15:**
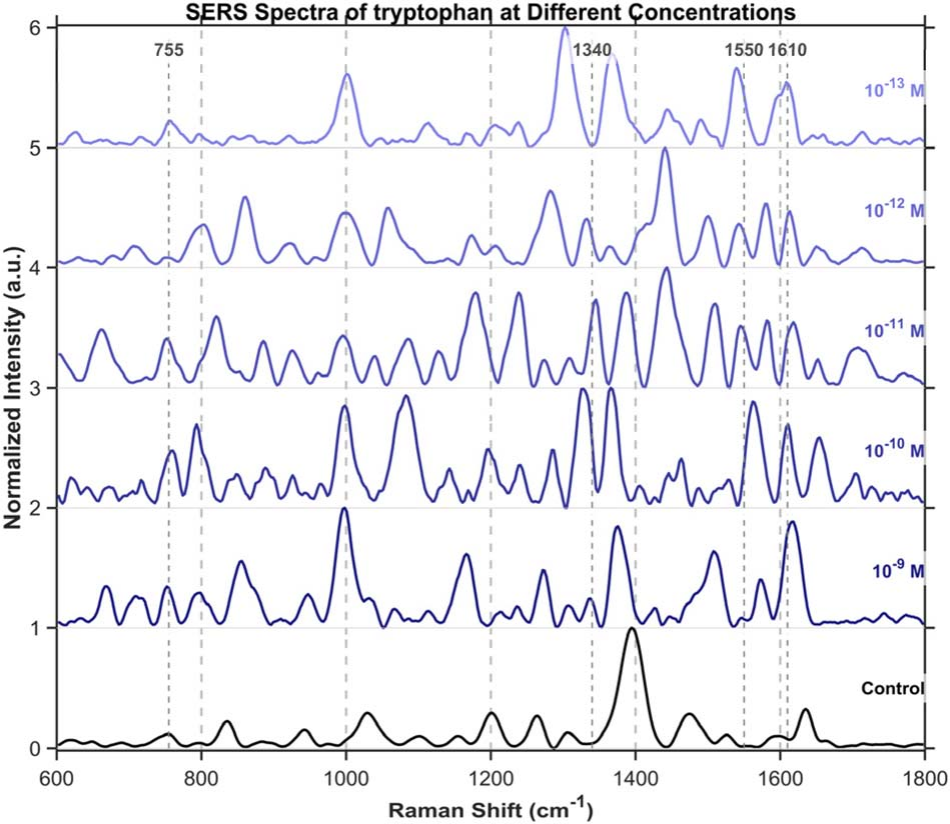
SERS spectra of tryptophan from 10^−9^M to 10^−13^M. Tryptophan's characteristic peaks were observed at 755 cm^−1^, 1340 cm^−1^, 1550 cm^−1^, and 1610 cm^−1^. These are attributed to tryptophan ring vibrations, CH_2_/CH_3_ twisting modes, amide II/ring vibrations, and aromatic ring stretching/C=C stretching modes, respectively.

**Fig. 16:**
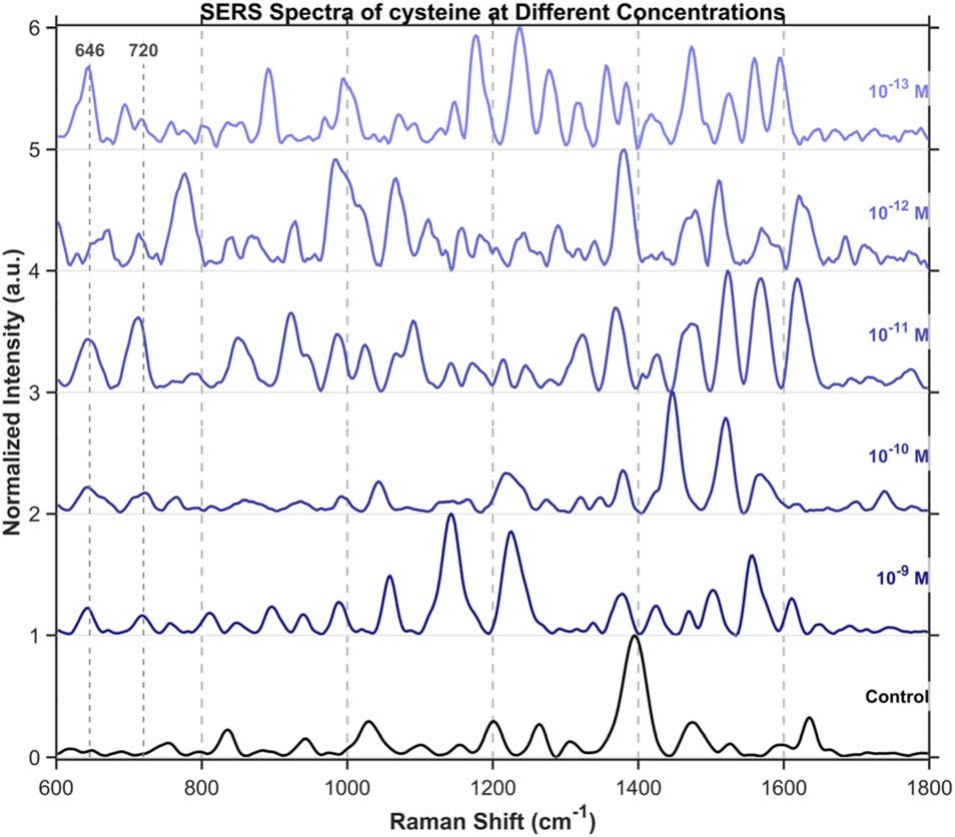
SERS spectra of cysteine from 10^−9^M to 10^−13^M. Cysteine's characteristic peaks were observed at 646 cm^−1^ and 720 cm^−1^. The band at 646 cm^−1^ is assigned to the C–S stretching mode and tyrosine ring vibrations, while the feature at 720 cm^−1^ corresponds to the C–S stretching mode of cysteine in the gauche conformation.

**Fig. 17:**
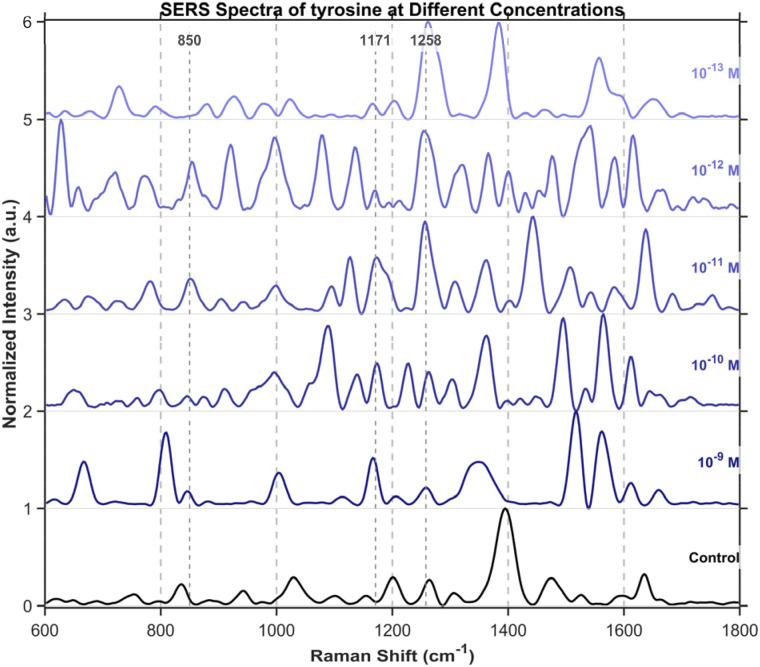
SERS spectra of tyrosine from 10^−9^M to 10^−13^M. Tyrosine's characteristic peaks were observed at 850 cm^−1^, 1171 cm^−1^, and 1258 cm^−1^. The band at 850 cm^−1^ is assigned to a tyrosine ring breathing vibration and forms part of its characteristic Fermi resonance doublet. The feature at 1171 cm^−1^ is attributed to a C–O–C stretching mode, while the peak at 1258 cm^−1^ corresponds to the amide III vibration, involving C–N stretching and N–H bending.

To further investigate the detection of small molecules, this study extended the scope of analysis to three specific amino acids, including tryptophan (Fig. 15), cysteine (Fig. 16), and tyrosine (Fig. 17), all purchased from Shanghai Titan Scientific Co., Ltd, and we performed analysis using the same method. Distinct characteristic peaks were also observed at concentrations as low as 10^−13^ M. The results confirm that the structure can effectively complete the detection of unlabeled small-sized molecules at extremely low concentrations.

### Single-spot SERS analysis

**Fig. 18:**
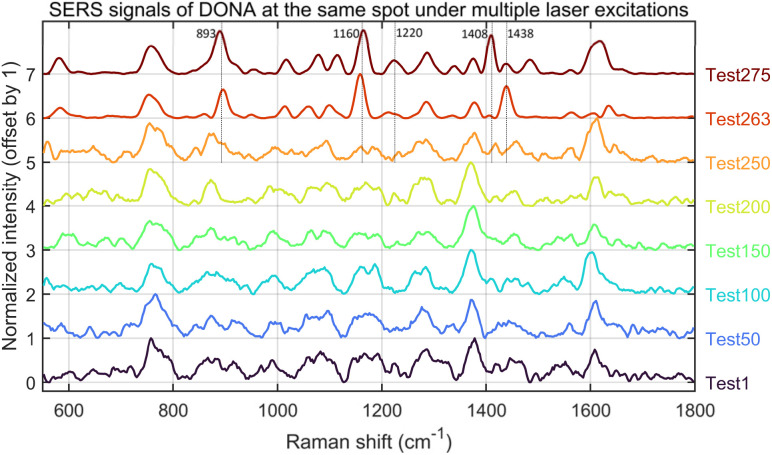
Time-dependent SERS spectral evolution. The data were acquired through continuous sampling at a single spot under 638 nm laser excitation (3.5% power, 2 s integration time). During the initial 250 seconds of irradiation, the SERS signal remained relatively stable. At the 263 seconds mark, a notable intensity enhancement occurred, with distinct peaks emerging at approximately 893, 1160 and 1438 cm^−1^, accompanied by a marked improvement in the overall signal-to-noise ratio. By 275 seconds, well-defined peaks had also developed at 1220 and 1408 cm^−1^, further confirming the spectral evolution under continuous laser exposure. Therefore, when performing SERS experiments with gold-assembled DNA origami, caution should be exercised regarding potential alterations in the DNA–gold bonding induced by prolonged laser irradiation.

Finally, we performed single-spot multiple SERS analyses on the DNA origami structure itself using a 638 nm laser at 3.5% power (1.18 mW) with a 2 s integration time per acquisition. The resulting data (after baseline correction with a structuring element size of 91; Fig. 18) were summarized by extracting key nodes of spectral variation. While sample drift may partially account for these changes, the signal evolution is also consistent with reported plasmon-driven interfacial processes.^42^ Under weak alkaline and oxygenated conditions, prolonged laser exposure may induce covalent Au–DNA bonding, thereby modulating the SERS response. Raman peaks at 893, 1160, 1220, 1408, and 1438 cm^−1^ are indicative of possible DNA–Au bond formation. This spectral signature is coincident with a substantial (over 5-fold) increase in SERS intensity, suggesting a direct link between interfacial bonding and plasmonic enhancement. We attribute this increase to a reduction in the hotspot gap, a mechanism consistent with the findings of Simoncelli *et al.*^16^ who achieved SERS enhancement by using laser irradiation to narrow the gap in DNA origami-templated structures. It should be noted, however, that the laser power and integration time required to induce such signal transitions varied considerably, even across different locations of the same sample batch.

Yeşilyurt *et al.*^43^ reported the formation of a carbonized DNA layer under prolonged laser irradiation, leading to spectral interference but no intensity enhancement—distinct from our observations. To our knowledge, no other studies have reported similar enhancement behavior, warranting further investigation.

## Conclusions

In this study, we have developed a highly sensitive and reproducible SERS platform by optimizing the structure and assembly process of a DNA origami nanofork antenna (DONA). A key innovation lies in our two-step strategy: assembling the complete DNA origami scaffold prior to conjugating with AuNPs. This post-synthetic conjugation approach not only preserves structural fidelity but also proves cost-effective by minimizing the consumption of expensive thiolated DNA strands—contrasting with alternative methods such as pre-functionalizing AuNPs with DNA^44–46^ or *in situ* synthesis of gold nanoclusters on DNA templates.^47^

By increasing the AuNP size to 80 nm and promoting the formation of higher-order multimers through additional thiolated staple strands, we achieved dense sub 2 nm plasmonic gaps that significantly enhance SERS intensity. This configuration enabled label-free detection of Rhodamine 6G (R6G) at concentrations as low as 10^−14^M, with characteristic Raman peaks clearly distinguishable. Furthermore, the platform successfully detected small proteins (*e.g.*, streptavidin, thioredoxin) and amino acids (tryptophan, cysteine, tyrosine) at sub-picomolar levels, demonstrating its broad applicability for ultratrace biomolecular analysis.

These results underscore the potential of DNA-origami-directed multimeric assemblies for next-generation SERS sensing. Looking forward, as DNA origami technology becomes more accessible and cost-effective, this platform can be extended to multiplexed detection in complex biological samples and integrated into portable diagnostic devices.

## Conflicts of interest

There are no conflicts to declare.

## Supplementary Material

RA-016-D6RA02441F-s001

## Data Availability

All data required to reproduce the findings of this study are provided within the paper and its supplementary information (SI). Supplementary information: the DNA sequences used in this study; control experiments with randomly aggregated gold nanoparticles for R6G detection; a detailed description of the baseline subtraction algorithm applied to the SERS data. See DOI: https://doi.org/10.1039/d6ra02441f.

## References

[cit1] Lin C., Li Y., Peng Y., Zhao S., Xu M., Zhang L., Huang Z., Shi J., Yang Y. (2023). J. Nanobiotechnol..

[cit2] Li J. F., Huang Y. F., Ding Y., Yang Z. L., Li S. B., Zhou X. S., Fan F. R., Zhang W., Zhou Z. Y., Wu D. Y., Ren B., Wang Z. L., Tian Z. Q. (2010). Nature.

[cit3] Kleinman S. L., Ringe E., Valley N., Wustholz K. L., Phillips E., Scheidt K. A., Schatz G. C., Van Duyne R. P. (2011). J. Am. Chem. Soc..

[cit4] Zhang Y., Zhao S., Zheng J., He L. (2017). TrAC, Trends Anal. Chem..

[cit5] Thacker V. V., Herrmann L. O., Sigle D. O., Zhang T., Liedl T., Baumberg J. J., Keyser U. F. (2014). Nat. Commun..

[cit6] Sharma M., Kaur C., Singhmar P., Rai S., Sen T. (2024). Nanoscale.

[cit7] Doppagne B., Neuman T., Soria-Martinez R., López L. E. P., Bulou H., Romeo M., Berciaud S., Scheurer F., Aizpurua J., Schull G. (2020). Nat. Nanotechnol..

[cit8] Nam J.-M., Oh J.-W., Lee H., Suh Y. D. (2016). Acc. Chem. Res..

[cit9] Fang W., Jia S., Chao J., Wang L., Duan X., Liu H., Li Q., Zuo X., Wang L., Wang L., Liu N., Fan C. (2019). Sci. Adv..

[cit10] Zhao X., Liu X., Chen D., Shi G., Li G., Tang X., Zhu X., Li M., Yao L., Wei Y., Song W., Sun Z., Fan X., Zhou Z., Qiu T., Hao Q. (2024). Nat. Commun..

[cit11] Gao Y., Zhang R., Cheng J.-C., Liaw J.-W., Ma C. (2013). J. Quant. Spectrosc. Radiat. Transfer.

[cit12] Li L., Yin J., Ma W., Tang L., Zou J., Yang L., Du T., Zhao Y., Wang L., Yang Z., Fan C., Chao J., Chen X. (2024). Nat. Mater..

[cit13] Schuknecht F., Kołątaj K., Steinberger M., Liedl T., Lohmueller T. (2023). Nat. Commun..

[cit14] Mostafa A., Kanehira Y., Tapio K., Bald I. (2024). Nano Lett..

[cit15] Niu R., Gao F., Wang D., Zhu D., Su S., Chen S., YuWen L., Fan C., Wang L., Chao J. (2022). ACS Nano.

[cit16] Simoncelli S., Roller E.-M., Urban P., Schreiber R., Turberfield A. J., Liedl T., Lohmüller T. (2016). ACS Nano.

[cit17] Acuna G. P., Möller F. M., Holzmeister P., Beater S., Lalkens B., Tinnefeld P. (2012). Science.

[cit18] Puchkova A., Vietz C., Pibiri E., Wünsch B., Sanz Paz M., Acuna G. P., Tinnefeld P. (2015). Nano Lett..

[cit19] Dutta A., Tapio K., Suma A., Mostafa A., Kanehira Y., Carnevale V., Bussi G., Bald I. (2022). Nanoscale.

[cit20] Li J., Wang W., Zhang H., Lu Z., Wu W., Shu M., Han H. (2020). Anal. Chem..

[cit21] Hemmig E. A., Fitzgerald C., Maffeo C., Hecker L., Ochmann S. E., Aksimentiev A., Tinnefeld P., Keyser U. F. (2018). Nano Lett..

[cit22] Prinz J., Matković A., Pešić J., Gajić R., Bald I. (2016). Small.

[cit23] Zhou C., Duan X., Liu N. (2015). Nat. Commun..

[cit24] Marras A. E., Zhou L., Su H.-J., Castro C. E. (2015). Proc. Natl. Acad. Sci. U. S. A.

[cit25] Zhan P., Wen T., Wang Z., He Y., Shi J., Wang T., Liu X., Lu G., Ding B. (2018). Angew. Chem., Int. Ed..

[cit26] Tanwar S., Haldar K., Sen T. (2024). J. Am. Chem. Soc..

[cit27] Tapio K., Mostafa A., Kanehira Y., Suma A., Dutta A., Bald I. (2021). ACS Nano.

[cit28] Mostafa A., Kanehira Y., Dutta A., Kogikoski S., Bald I. (2023). J. Vis. Exp..

[cit29] Kanehira Y., Kogikoski S., Titov E., Tapio K., Mostafa A., Bald I. (2024). ACS Nano.

[cit30] Kooij E. S., Ahmed W., Zandvliet H. J. W., Poelsema B. (2011). J. Phys. Chem. C.

[cit31] Chen J., Huang Y., Kannan P., Zhang L., Lin Z., Zhang J., Chen T., Guo L. (2016). Anal. Chem..

[cit32] Lin D., Wu Z., Li S., Zhao W., Ma C., Jiang Z., Zhong Z., Zheng Y., Yang X. (2025). ACS Nano.

[cit33] Hanske C., González-Rubio G., Hamon C., Formentín P., Modin E., Chuvilin A., Guerrero-Martínez A., Marsal L. F., Liz-Marzán L. M. (2017). J. Phys. Chem. C.

[cit34] Storhoff J. J., Lazarides A. A., Mucic R. C., Mirkin C. A., Letsinger R. L., Schatz G. C. (2000). J. Am. Chem. Soc..

[cit35] Dinkel R., Jakobi J., Ziefuß A. R., Barcikowski S., Braunschweig B., Peukert W. (2018). J. Phys. Chem. C.

[cit36] Mayr F., Zimmerleiter R., Farias P. M. A., Bednorz M., Salinas Y., Galembek A., Cardozo O. D. F., Wielend D., Oliveira D., Milani R., Brito-Silva T. M., Brandstetter M., Padrón-Hernández E., Burgholzer P., Stingl A., Scharber M. C., Sariciftci N. S. (2023). Anal. Sci. Adv..

[cit37] Chen H., Xu W., Broderick N. G. R. (2019). Appl. Spectrosc..

[cit38] Campione S., Adams S. M., Ragan R., Capolino F. (2013). Opt. Express.

[cit39] Tira C., Tira D., Simon T., Astilean S. (2014). J. Mol. Struct..

[cit40] Le Ru E. C., Etchegoin P. G. (2006). Chem. Phys. Lett..

[cit41] Gomez-Cruz J., Bdour Y., Stamplecoskie K., Escobedo C. (2022). Biosensors.

[cit42] Su Y.-Q., Liu J., Huang R., Yang H.-T., Li M.-X., Pang R., Zhang M., Yang M.-H., Su H.-F., Devasenathipathy R., Wu Y.-F., Zhou J.-Z., Wu D.-Y., Xie S.-Y., Mao B.-W., Tian Z.-Q. (2023). J. Phys. Chem. Lett..

[cit43] Yeşilyurt A. T. M., Wu X., Tapio K., Bald I., Huang J.-S. (2023). J. Am. Chem. Soc..

[cit44] Xie M., Jiang J., Chao J. (2023). Sensors.

[cit45] Zhao Z., Jacovetty E. L., Liu Y., Yan H. (2011). Angew. Chem., Int. Ed..

[cit46] Schreiber R., Santiago I., Ardavan A., Turberfield A. J. (2016). ACS Nano.

[cit47] Rai S., Kaur V., Kaur C., Sharma M., Sen T. (2025). Nanoscale.

