# Supplementary material for: DNA origami-engineered gold nanoparticle multimers for ultrasensitive, label-free SERS detection of small molecules and biomolecules
Source: RSC Adv. 2026 Apr 22;16(23):21266–76. doi: 10.1039/d6ra02441f (PMC13102148; doi:10.1039/d6ra02441f)
Supplement: RA-016-D6RA02441F-s001 [file RA-016-D6RA02441F-s001.pdf]

Supplementary Information (SI) for RSC Advances.
This journal is © The Royal Society of Chemistry 2026

Electronic Supplementary Information (ESI) for "DNA Origami-Engineered Gold Nanoparticle Multimers for Ultrasensitive, Label-Free SERS Detection of Small Molecules and Biomolecules"

Yaosen Wang,[a] Yanlong Cai,[b] Huaizhou Jin[b*] and Shangzhong Jin[a*]

[a]College of Optical and Electronic Technology, China Jiliang University, Hangzhou 310018, China.

[b]Key Laboratory of Quantum Precision Measurement, College of Physics, Zhejiang University of Technology, Hangzhou, China.

* Corresponding author: jinsz@cjlu.edu.cn;

1. Table of strands

Core staple1	AAGGGACTGGCGCGTCGCCGCGAGCGAGAAAGGA
Core staple2	TGACCTCCGCTACAGTAGCCGGTCAAGA
Core staple3	TTCTGCGAAGAAAAATAACAGAGGTGAGGCCCATTTACATTG
Core staple4	CTACAATAATGAGTGACCCTGATTAGAGCCCGTAAT
Core staple5	TTCATTGCAATACTTTTAGCGAAAATCACC
Core staple6	AACCGGCCTCAGGAAGATCCGTTGTAA
Core staple7	ATAGAAACCAGGCAAAGCGAAAGGGGG
Core staple8	GTCATAGAATGGGATAGGTCACGTTGGTCGTAATCA
Core staple9	AGCCCCCGATAACAGTATGAGCAATTTTATTTTC
Core staple10	GTTAGCGTAACGACGTTGAAA
Core staple11	AACAAGCAAGCCAGAGCCT
Core staple12	GGAACGGTACGTCTGTCGATTGTTCCAGAAG
Core staple13	ATAAGAGCAAGAAAAATAATAATGTACCGACAGTC
Core staple14	TTATCAACAATATTGTTTA
Core staple15	TTAGTACCGACTTTATACATTTCACGACCAGTAATATACCGAACCTCAAA
Core staple16	AACGACGAGCGCTGCGCAGCGAAATTGACGGTAAA
Core staple17	ATTAATGAATCCGGAATAAATACCCATCCTAATT
Core staple18	GAACAACGTATCGGAGGGAGTGAGGCT
Core staple19	GCTGCAAGCGACGTGCTTTC
Core staple20	GCAAAAGAAATACCCAAACAAGAGGATTTAGAAGTTGCTGAACGAACCA
Core staple21	AGCTGGCGCCAAGTGTTTTT
Core staple22	GCAAATCAGATATCTTACC
Core staple23	AGAATAACATAAAATTCTGTCACAACATCACTGCC
Core staple24	ATAATAACTCCAAAGCGGGATACGAGGGTAGCA
Core staple25	GAGCGGACGAACGTACCGCCAAACCCTTCTGACCTTTAATGCAATCAAC
Core staple26	CGCATAGGGTCAATGTATTAAACCAGGC
Core staple27	CGAGTGTACACGGATATATTCAGTAATCATTTCATTATAACAACATTTA
Core staple28	AACCTCCCGACTTTGCACC
Core staple29	AGCGAACGGCCGGACCGGTTGAAATTCG
Core staple30	AACATCACAATATTTATTAATCTAACAACTAATAGGTTGGCAGCGAACT
Core staple31	CCCGCCACCACATAATCTCAGACTGACAGAAGAGCCATGGCTTAATTCG
Core staple32	CATTAAATTTTTGTCAATCATACGGAATGGTGGCA
Core staple33	TCCATCAAACGGAGAGCTGGAGCTAATTCGCGTCTGG
Core staple34	GGTAACGCCGGCGCTAGGGC
Core staple35	AAAATAATCATAGCTTCCTTAGAAAAACCGCTAA
Core staple36	TATTAAACCAAGCAGTTAC
Core staple37	TAACCTTATTAACAAAGCTGCTCTCATTACTTTAACGTATTTCGGA
Core staple38	CTTAAACCATTTCAACAGTTTCAGCGGATTCGAGGTATTCGGTTTTTCA
Core staple39	CGCCACCCTCAGACTAAAGTAACAACCTACCGAT
Core staple40	AAGAAGTGCAACACCGCCTCCCTCAGATTATTAGCTCGG

Core staple41	GAAAGGGGACGACGACAGTTGCCAAGC
Core staple42	TATCAAATACATTTTTCGACAATACTCAGGAGGT
Core staple43	TAGGAACGCTGAATGGCTTGCTTTATCAGCTTGCTGTGAGAATTCTGTA
Core staple44	CCAGCAGCCAGTCATGACGCTCTAAAACTACCAAGTCGCCTGGAAC
Core staple45	CCAGTATATCACCAAAAATCAAGAAAACGAGAATGTCCACGCCATTCAAA
Core staple46	AGAAGAGCGTCGCTGGCCCACGGGTCGA
Core staple47	GCTCATTTTTTAACGTAATCGGCATGATCGTAGAA
Core staple48	ATAAGGGCCGGAAACTTAGTTAGTTGCAGGAAAATC
Core staple49	CCTCAGAACCCACCAGACTAACGGACCAGTC
Core staple50	GAAAAACAAATCAAGGTAAATTTATACATTTAACAAAAAGGTTGAGG
Core staple51	GCATCTATCACACTATTAGCCCGAATG
Core staple52	ACGTCAAAAATGAACGCGCCT
Core staple53	GAAAAATAATATTAAGAAA
Core staple54	AGCTATAATGAATTTAGAAAG
Core staple55	GAATTGAGTTAAGAGAACTGTAAAACTATGATAT
Core staple56	TTAATATTTTGTTAATAATCAAACCGAGCGCAAAG
Core staple57	CTGGTCAATTAGAGCTTTGCCATTATCAATCAATATATTAGGATTAGC
Core staple58	CGGGAGAAGCCTTTATTTCAAAACTCCAACAGGTCAGGATTA
Core staple59	ATACAGGTGCCTTGCAAGAACGACCAGG
Core staple60	ATATCAGAGAGATACCTTATTAGAATCGTGATAAA
Core staple61	TAGGAATCATTAACGAGCG
Core staple62	TCAACCGAGACTTCTATTGACGGAAATTTAGCAAATAAGACTACCCACAA
Core staple63	GCTTTAAATGTTTAGTTTTCAGTTTGCC
Core staple64	CGGCCTTGCTTAACATTTCTAAAACGGGAGGTTT
Core staple65	ACGGCTACATAAAGGCTAGCATTCTTAATTCCATGTACCGTAAC
Core staple66	TGAGAAAATTTTTAACAAAAGGGCGACAAAAGAAAGAAACGCCAATGAA
Core staple67	TCAAGAAACAAAATCCACACCACTATGG
Core staple68	ATCAAAACGCCGCCCCTCTACGTTAATATAATGCAAGTAAGATTTGCC
Core staple69	ATGCCTGCTGATCGGCAAAA
Core staple70	CTGTTTGGATAGGGCGGCTTAGACGCTG
Core staple71	CGCGAGCTCGTCTTTATTGCGA
Core staple72	AATTACTATAAGTCCTGAA
Core staple73	TTGCACGTAATCCTCATCACGTAGTAAT
Core staple74	GGAACCCTCGAGAGGCTTAGCGTCAATCCCCAGTCAGAAGAGAT
Core staple75	GACAAAGTAACGAAGGCACCAACCAATCGTCCATATA
Core staple76	TTGCTTTAGGAGGCTAAAGAAAAATTAT
Core staple77	AGTTGAAAGGAGCATTTAAAAGAAACCATGGATTACATA
Core staple78	CAGCTACGCGAACAAAGTTTGAGGGTAATATAAGAATACGTGGCACA
Core staple79	GAAGGAAAGGGAGAACCAAGTTAACAAA
Core staple80	ACAGTAGTTGGGAACTATTATCTCAAAT
Core staple81	GCCCGAATTCTAGCATGAACGCAATAGGAACG
Core staple82	GTGTGAAATTATGTTCAGCTAA
Core staple83	ACAGTTAGCCACCCATAGCCCTGCCGTCTCTGAAACCCTGCCGGGT

Core staple84	TTATAAATCAACTATATCATAGCGATTCCAAGAA
Core staple85	TTCACAAAGCGCAGCAACTTTGAATAAGGCTTGCCATCTTGAAGTAAC
Core staple86	GGGACGACGTTCATCAGTTGAGATTATTACACCCTCAAATCCTCAT
Core staple87	AATTGCGTAAATCAGTAGCACATGAAACCATCGATTTTCGGTTGGGTAAT
Core staple88	CAACGCGCAGTATGTATTCATTAGAGGAA
Core staple89	TGAGGAAGTTTCCAACGCATATTTTAAACATTTCG
Core staple90	CCTAGATGGGCGCATCGTACCGGGTAC
Core staple91	GCCTTAAATCAAGATTAGT
Core staple92	GAGTAACAACGGAAGCAAAAAGGTATTCTTA
Core staple93	GTGGCTGGCCCTCTTTTCCGCGCGGCAGTCGGGCTCACACAAACGG
Core staple94	CCTGATTACTCGTAAAAAACGCATTCTGGCCAACACTAAAACTCAATAT
Core staple95	GAGGATCCACAGACGGGCAA
Core staple96	ACATATATTCAACCGCTTCAA
Core staple97	AGCAACAAAGTCAGAGGGTAATTGTAGGCAGTGAGAGTGGTAGCT
Core staple98	GTCCAAATCAGGACAGATGAACGACTGACCCTATTATGAGAGGGTT
Core staple99	CGGGCACCGCTTCTGGTGCGCGATTAA
Core staple100	GGTAAAATACGTAACAATGACACGGTGTATTTAGT
Core staple101	TTTAATTTAAAGGACCAGACGTTAGTAATTTTCATTTGGGG
Core staple102	CCTCATTTTCAGGCAACATGACCGATATGAATTT
Core staple103	AGCATAAAGCTAGCGGATGGCTTA
Core staple104	TCCTGAATAAACCTACTACTTCTCACCGAGGAGT
Core staple105	CATAGCAGCACAGCAAAAAAGCCATATAAAGGAGAATT
Core staple106	GGCCTCTTGCAGAACTCAAA
Core staple107	TGCTATTTGTATCTTTAGGAATTATGGCTA
Core staple108	CAGAACGCTTAATGTTGAATAACAATAA
Core staple109	GATAGCCGAGATAGGCCATTGCTTTGATCAAATTATATTTTAGACA
Core staple110	TAAGCAGATAGCGATTGTAGTAATGTGTAGGTATTTAAATTATGGTT
Core staple111	ATAGAAAATTCAGCAATGCCT
Core staple112	GAAAGACAGCATCGGACGTCACCACTCAATTCTACTATTCGTCA
Core staple113	CCTTCCTCATTGCCAGGCATTTTGAGAA
Core staple114	GTGCGTGCATCTGCCAGTTAGGTCGAC
Core staple115	GAGATTCGCCATTCAGGCTCGCTATTA
Core staple116	TGTCTTTCCTTAACAGCCA
Core staple117	GCAGATTCAAAGATAACTAAAGCATCACCTATTGATTCCCAA
Core staple118	GGGGTTTTGATGATGTACACTCATCTTTGAATCCGCCGCAGACGCTGGC
Core staple119	AAATCACAATTCGAGATTGAGGGAGGGAACATAAAACCCAAACCCAATA
Core staple120	GTGTCGAACCCCCAGTGTATCACCGTCAGCTCAGTGAGGCTGGCCGTATA
Core staple121	CCGCGGAATAAACCAAAATAGCGTTTACCAAACCAGATCAGAGCCA
Core staple122	CAAATGGTTGCTAAACAACACAGTGCCATTAGATATATGCAAGCCACCAC
Core staple123	AACAGTTAATGCCCATGAAACATAAGGATAAATT
Core staple124	TTGACCACGCTGAGATTAACACCGCCTGAGCTTGAATCGCCCTTAAACG
Core staple125	AAGAGTACTGACGAAGATGGTTGGGAA
Core staple126	AAGAGTCGGGCGATATTAATTAACCTTGCTTCTGTTTTCATTCGGCGA

Core staple127	AGGACGTTTAATTTTCTCTGATTTGATG
Core staple128	CAGTAGCGTAGCGCGACTGGATTTGCAA
Core staple129	CGATTTTGAGAAAAAGATAAACACGGCCAAACCA
Core staple130	TGCAGAAAATAGCAGCCTTAAACAACGTTATCCGAAA
Core staple131	AGCATGTAGAAATATCCCA
Core staple132	ATATTTTTGAGAGGAAGG
Core staple133	GTTGTTAGAGCAGGCCAAGCGGACTGGGCGCCAGGG
Core staple134	CCAGGTAGATTTTAAGAACTGGCGTGAATTAAGCCAGTGGTAATAA
Core staple135	TAGATAACCCTCAAATCGCCATTAAAAAAAAGGGACTCATGGAATACA
Core staple136	AGAGCAACTGTTGGGAAGGGCGATCGG
Core staple137	ATTAATAGCGTCATAGATTGCTCGTCATC
Core staple138	AATCGGTTGTACCAAAAACATTTTTGATAAGAGGTCATTTTT
Core staple139	GTTTTGTGAAAAGGTGGCAAGACAGCCCTCATA
Core staple140	CCATCAAAAAAAACAAGACCAACATGTAATTAGCGCTA
Core staple141	AATTTGCTAATATGTGTAATGGATTAGAGCGGAG
Core staple142	TCATCAACATTGGGGTGAAGAGAAACGCTCA
Core staple143	TGGGATTTCAATAATGTAGCTGATAGCAAGCCCAA
Core staple144	TATATTGTCACCACATTACCTATACAAAAAGTAAACAGGGAAGCG
Core staple145	GCAAGTGTAGTTTCAATTCAGATGGCGCCCAATA
Core staple146	CGGATTGACCGTCTGTTTC
Core staple147	AATACATAGGTAAAAAATATCGCGTTTTCATCAATAGCATGTTAAATCA
Core staple148	CAGGTCAGACGTTCCAGGGGCTTGGAAACAC
Core staple149	TCAATTGCGTAGTAACAAGGCGAATGATGAACCTTTTTAGT
Core staple150	ACCGGAAGCACGCAAGGATA
Core staple151	TTAGTCTGAAAGCGTCCAGAACTTGCCTTAAAAGAGCCAGAAGCTA
Core staple152	TATGCGTATTAGCACGGTTTGCGTATCATTGCGCTACGAGCCCCGTCGG
Core staple153	TTAATGCAAGATTAAAAGGTGAATTATCATTTAA
Core staple154	TCGCCATACCGTCACGGATTGATAAATA
Core staple155	AACGCCAACCATCAAGCATTGACAGGATTCAATAGACCTTTTCGAGAA
Core staple156	CTCGAATTGTGTCGTGCCAG
Core staple157	ACGGCCAGATGTGGACTCCA
Core staple158	AACTGAACACCCTGCAGTAATCCAGGCTATCAGGTGTAGCCAGCTT
Core staple159	TATTCTAAGAACAATTTTA
Core staple160	GGATAAGGGAATAGGCGATTAGAAAGAG
Core staple161	GAATAATTTTCAGATTAAGGTTGGGCCAATCGCTTCTGATGATAA
Core staple162	ATAGCAATAGCTATAGAAGGAGAAAAGCAAAAGGG
Core staple163	TATGACCCTGTAATACTTTTGGAGAGTACCTTTAATTGCTCC
Core staple164	CTAAAAGCAATACTTCAACAGGTTAAATCCCGTCAA
Core staple165	TACCAGCGGAATAAAGTTACCCTTACCGAAGCCCTTTTTAAGAAAAG
Core staple166	ATTCAACAAACGAAACCACCACTTGATA
Core staple167	TTAGAATCAGCAGGTTTGGGTTAGGAAGGCTTAT
Core staple168	AGTGCCCTCATGGCTATTTACCGATTGGCCCAGAGCTCAGCCGCCAC
Core staple169	TGGTTTTTGAGAGAATTTCATCAAGACAAAGAACGTAACCTCTTGAGT

Core staple170	CGCCACCTTTCATTCTGAAATGGATTGATGCCACTCA
Core staple171	ATTCTCCGTGGGAAATTCCACCAGACGAAGTATCA
Core staple172	AGAGGGGTTTGCCCCGAATTACAGAGACTTGAATTT
Core staple173	ACGTGGTATTTTTTGTACGTGAAACCAGTTTGGAAC
Core staple174	AACTTTTAGCCCCCTAGGTCTGGAGGCATGATACAT
Core staple175	CAAAGGGCGAATCCTTGAATCAATCCGCACTCAT
Core staple176	CGGATTCAGACTCCAACGAGGGACCTGC
Core staple177	ATCTTTTCCGGAACTATCATAATACCAC
Core staple178	CTGTAGCATTCCCTCAGCACTCCAAAAAAAAGGTTTTTTCATCTAAA
Core staple179	ATTTTTGAGCAAAGCCGACTTTCAAGTTTG
Core staple180	ACACCACGCCAAAGGAACCCT
Core staple181	CATTAGACGGTACCGACTAAAGTGTCACATT
Core staple182	AGTCACGAGCGGAACCCTAA
Core staple183	ATCACTCCAGCCAGCTTTCCAGGGTTT
Core staple184	TGATTGCCCTTTAATGGATGCTGAAATCAATAAT
Core staple185	TATTATTCCTGCAAATTTATATAAAAGAATAAAG
Core staple186	CGCTTTCGGAGAGGAGCGTTAAATAAGACCTGTTTCGACAATTACAGAG
Core staple187	CAGAGTGTACAATGGAAACAAATAGAGCCGCGCCA
Core staple188	CCAGTACAAACTACAACGC
Core staple189	TCTTTCCGTAAGAAGATTATTCACGGTCACACGT
Core staple190	AACGCTACCAATATACAGATTTTAGCGGGATCCT
Core staple191	AGTTGCGGGTCAGTAGCCAGCAGCAAATCGAGTAGCTGGAAGCTCAGAAC
Core staple192	TCAGTGAGGCGAATAATTTTTGCGGAGGCGTTTT
Core staple193	AGCAAATATTTAAATTACAGGAACGAACAAGTTTATTTGTCACAATCA
Core staple194	ACAACTTTGGGAGATTTGTATCACGCGAAATATAAGTTCAGAAC
Core staple195	TTGAGGACTAAAGACTCGCTGAATAATGCCCTGTTT
Core staple196	CATATATAAGATTCCCCAAAAGTAAACG
Core staple197	AGGGAAGAAGTAACCACGCGCAGGTACCTTTTACATCACAGAAACGATTA
Core staple198	AGTAAAACAGTTCGGTCTTTAGCTAACTAAAGCCTAAATGTGAGC
Core staple199	ACTGAGTATAGTAGCGCTTTTAGGAGCC
Core staple200	GGTGCCGGGAAAGCTGAATTAACAAACA
Core staple201	ATCCAAACCGACCGTGCCTAAATTCACCGCTTCC
Core staple202	TCCATGTACCGTTGGCAATTCTCATATT
SH_103	HS-AAAAAAAAAAAAAAAAAAAAAAAAAGCATAAAGCTAGCGGATGGCTTAAACAACAAC AACAACAACAACAAC-SH
SH_150	HS-AAAAAAAAAAAAAAAAAAAAAAAAACCGGAAGCACGCAAGGATAAACAACAACAACA ACAACAACAAC-SH
SH_56	HS-AAAAAAAAAAAAATTAATATTTTGTTAATAATCAAACCGAGCGCAAAGAACAACAACAA C-SH
SH_65	HS-AAAAAAAAAAAAACGGCTACATAAAGGCTAGCATTCTTAATTCCATGTACCGTAACAA CAACAACAAC-SH

Supplementary Table 1. DNA sequences used in the experiments. The DONA structure was assembled from 202 staple strands and the M13mp18 scaffold strand. Four of these staples were

dual-end modified with thiol groups to replace their original counterparts for conjugating gold nanoparticles.

2. R6G detection for random AuNPs

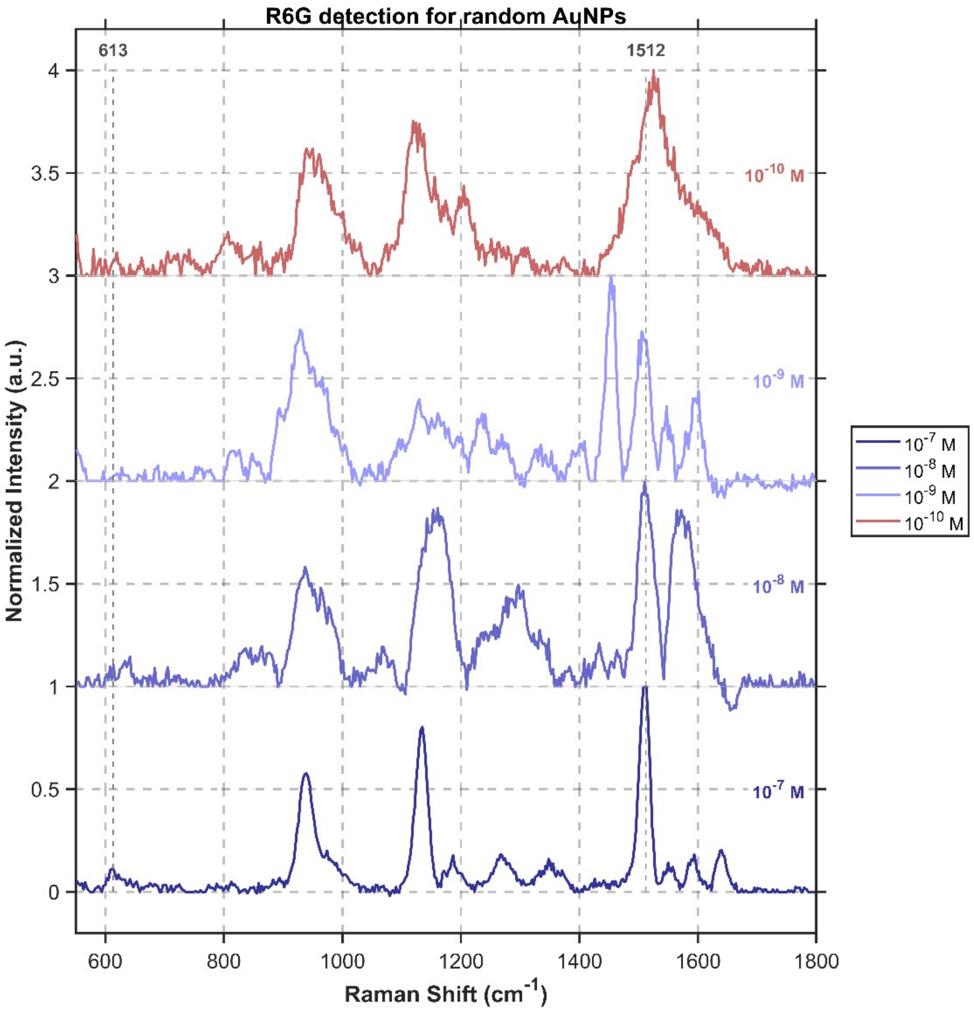

Supplementary Figure 1. The optimal detection results for R6G concentrations ranging from 10^{-7} M to 10^{-10} M were obtained by depositing a 1:1 mixture of the AuNP solution and the R6G solution, followed by SERS measurement using a 638 nm laser at 1.18 mW power with a 1 s integration time. In contrast, detection using randomly aggregated AuNPs without the DNA origami scaffold showed limited performance: the detection limit was insufficient, the substrate exhibited poor reusability, and it was difficult to locate suitable scanning areas within the field of view.

3. Introduction to the Baseline Correction Method

Three-Step Baseline Correction and Spectral Preprocessing Pipeline

Input:

- Raw spectral data: raw_spectra $\in \mathbb{R}^{m \times n}$, where m is number of spectra, n is number of wavenumber points
- Parameter structure: params (containing various thresholds and settings)

Output:

- Corrected spectra: corrected_spectra $\in \mathbb{R}^{m \times n}$
- Baseline estimates: baseline1 $\in \mathbb{R}^{m \times n}$, baseline2 $\in \mathbb{R}^{m \times n}$
- Normalized spectra: normalized_spectra $\in \mathbb{R}^{m \times n}$

Procedure:

1. Initialization and Preprocessing:

1.1 Define fingerprint region: wavenumber_range $\in$ [600, 1800] cm^{-1}

1.2 Extract fingerprint region data: fingerprint_data ← raw_spectra(fingerprint_region)

1.3 Optional: Apply Savitzky-Golay filter for data smoothing

2. Three-Step Baseline Correction (executed for each spectrum):

for i = 1 to m do

spectrum ← fingerprint_data(i, :)

Step A: Morphological Baseline Correction

Input: signal ← spectrum, params

Process:

a. Set structuring element length: L ← if params.use_fixed_length then 81 else 51

b. Initialize: baseline_prev ← signal, baseline_current ← signal

c. for iteration = 1 to params.max_iterations do

i. Create linear structuring element: SE ← strel('line', L, 0)

ii. Morphological opening: opening_op ← imopen(baseline_prev, SE)

iii. Morphological closing: closing_op ← imclose(baseline_prev, SE)

iv. Compute average: avg_op ← (opening_op + closing_op)/2

v. Update baseline: baseline_current ← min(signal, avg_op)

vi. Smooth baseline: baseline_current ← gaussian_smooth(baseline_current, window_size=L/2)

vii. Check convergence:

ifiteration>1and$\sum$(baseline_current-baseline_prev)^2/$\sum$(baseline_prev)^2 < params.convergence_threshold

then break loop

viii. baseline_prev ← baseline_current

end for

d. morphological_baseline ← baseline_current

e. preliminary_corrected_signal ← signal - morphological_baseline

Step B: Optimized Piecewise Baseline Correction

Input: signal ← preliminary_corrected_signal
Process:
a. Smooth signal: smoothed_signal ← moving_average(signal, window_size=5)
b. Find local minima: [minima, min_locations] ← findpeaks(-smoothed_signal)
c. Ensure boundary points included:
if min_locations(1) ≠ 1 then add smoothed_signal(1) at position 1
if min_locations(end) ≠ n then add smoothed_signal(n) at position n
d. Linear interpolation: piecewise_baseline ← interp1(min_locations, minima, 1:n, 'linear')
e. Smooth piecewise baseline: piecewise_baseline ← moving_average(piecewise_baseline, window_size=11)
f. Ensure baseline doesn't exceed signal: piecewise_baseline ← min(piecewise_baseline, smoothed_signal)
g. secondary_corrected_signal ← signal - piecewise_baseline
h. Ensure non-negative: secondary_corrected_signal ← secondary_corrected_signal - min(secondary_corrected_signal)

Step C: Normalization and Thresholding
Process:
a. Max-value normalization:
if max(secondary_corrected_signal) > 0
then normalized_signal ← secondary_corrected_signal / max(secondary_corrected_signal)
else normalized_signal ← secondary_corrected_signal
b. Intensity thresholding:
threshold←params.normalized_intensity_threshold×max(normalized_signal)
normalized_signal(normalized_signal < threshold) ← 0
c. Forced zeroing:
low_threshold ← threshold × 0.5
normalized_signal(normalized_signal < low_threshold) ← 0
d. Optional: Apply Savitzky-Golay filter to normalized signal

Save Results:
corrected_spectra(:, i) ← normalized_signal
baseline1(:, i) ← morphological_baseline
baseline2(:, i) ← piecewise_baseline
end for

3. Calculate Average Spectrum:
average_spectrum ← mean(corrected_spectra, dimension=2)
Repeat Step C normalization and thresholding for average_spectrum

4. Output:
Return corrected_spectra, baseline1, baseline2, normalized_spectra
